# GWAS identifies regulators of seed and leaf morphology in common bean, revealing OFP5 as a major seed size determinant

**DOI:** 10.1111/tpj.70909

**Published:** 2026-05-06

**Authors:** Julia von Steimker, Mustafa Bulut, Gregory Pozhvanov, Susan Bergmann, Regina Wendenburg, Yuming Sun, Feng Zhu, Alice Pieri, Giulia Frascarelli, Laura Nanni, Elisa Bellucci, Elena Bitocchi, Roberto Papa, Alisdair R. Fernie, Saleh Alseekh

**Affiliations:** ^1^ Max‐Planck‐Institute of Molecular Plant Physiology Am Mühlenberg 1 14476 Potsdam‐Golm Germany; ^2^ Program Center MetaCom Leibniz Institute of Plant Biochemistry Weinberg 3 Halle (Saale) 06120 Germany; ^3^ Komarov Botanical Institute Russian Academy of Sciences Saint‐Petersburg Russia; ^4^ Jiangsu Key Laboratory for Conservation and Utilization of Plant Resources, Institute of Botany Jiangsu Province and Chinese Academy of Sciences Nanjing 210014 China; ^5^ Key Laboratory of Horticultural Plant Biology, National R&D Center for Citrus Preservation Ministry of Education, Huazhong Agricultural University Wuhan 430070 China; ^6^ Department of Agricultural, Food and Environmental Sciences Polytechnic University of Marche Via Brecce Bianche 60131 Ancona Italy; ^7^ Center of Plant Systems Biology and Biotechnology Plovdiv 4023 Bulgaria

**Keywords:** *Phaseolus vulgaris*, leaf and seed phenomics, GWAS, seed size, PvOFP5

## Abstract

*Phaseolus vulgaris* (common bean) is one of the most economically important members of the Fabaceae family and a crop of high nutritional value. Seed and leaf morphological traits are key determinants of yield, shaped by genetic and environmental factors. In this study, we characterized a diversity panel of 434 *P. vulgaris* accessions for morphological and colorimetric traits. Using genome‐wide association studies based on both single‐nucleotide polymorphisms and structural variants, we identified 73 high‐confidence quantitative trait locus (QTL). In total, we present 114 candidate genes within 25 quantitative trait loci linked to seed and leaf morphology and color, including an *OVATE Family Protein 5* (*PvOFP5*), which was functionally validated as a key regulator of seed size in common bean. We employed a heterologous approach by overexpressing the inferior (Mesoamerican) and superior (Andean) alleles of *PvOFP5* in *Arabidopsis thaliana* wild‐type and knockout lines, and confirmed the key role of PvOFP5 in determining seed area. This work provides a comprehensive atlas of genetic associations for bean morphology and color, and highlights PvOFP5 as a promising target for marker‐assisted breeding aimed at optimizing seed size.

## Significance Statement

Seed and leaf phenomic traits, including morphology and spectral features, play key roles in consumer preference and plant performance. Here, we conducted a genome‐wide association study of 71 phenomic traits, identifying 73 high‐confidence quantitative trait locus and 114 candidate genes. Focusing on PvOFP5, we demonstrate its role as a key regulator of seed size through functional analysis of inferior and superior alleles in a heterologous system.

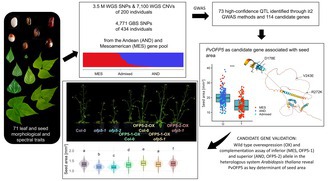

## INTRODUCTION

The common bean (*Phaseolus vulgaris*) is one of the most important legume crops and the most widely consumed by humans. It is a diploid (2*n* = 2*x* = 22), predominantly self‐pollinated species and one of the five domesticated species within the *Phaseolus* genus (Bitocchi et al., 2017). It holds significant economic and nutritional value worldwide (Bulut et al., 2023; Cronk et al., 2006; Hayat et al., 2014; Murube et al., 2021). Beans are consumed both as immature pods (snap beans) or mature dry seeds, and both are a rich source of proteins, complex carbohydrates, dietary fiber, minerals, and bioactive phenolics (Hayat et al., 2014). Due to their low carbon and water footprints and ability to fix atmospheric nitrogen, beans also contribute significantly to sustainable agriculture (Uebersax et al., 2022). In 2023, global dry bean production reached 28.5 million tonnes, with an export value of 5 million USD, with the top five producers being India, Brazil, Myanmar, China, and the United States (FAO, 2025). Common bean exhibits substantial morphological diversity, particularly in seed shape, size, weight, and color‐related traits (García‐Fernández et al., 2021; Giordani et al., 2022; Jurado et al., 2024). Seed morphology is important to both consumer preferences and agronomic value. These traits influence market class, processing quality, cooking time, water absorption, and nutritional composition (Berry et al., 2020; Vidak et al., 2022). For example, the seed coat not only determines color and texture but also contributes to mineral content (Blair et al., 2013).

Two independently domesticated gene pools, the Andean and Mesoamerican, have shaped the global diversity of common bean (Bellucci et al., 2023; Bitocchi et al., 2012; Cortinovis et al., 2024; Gepts et al., 1986; Singh et al., 1991). These pools differ significantly in seed traits such as size, color pattern, and storage protein composition (Murube et al., 2021). Their hybridization in post‐domestication breeding has introduced broad phenotypic variation in plant and seed morphology, especially in European cultivars, which often carry introgressions from both pools (Angioi et al., 2010; Bellucci et al., 2023; Campa et al., 2018; Santalla et al., 2002).

This phenotypic diversity has enabled genome‐wide association studies (GWAS) and quantitative trait locus (QTL) mapping approaches to uncover the genetic basis of important agronomic traits (Alves et al., 2024; Blair et al., 2009; García‐Fernández et al., 2021; Giordani et al., 2022; Izquierdo et al., 2023; Jurado et al., 2024; López‐Hernández et al., 2023; Mir et al., 2021; Murube et al., 2020). Recent GWAS have identified numerous QTL associated with seed morphological traits (Alves et al., 2024; Giordani et al., 2022; Jurado et al., 2024), pod morphology and color (Di Vittori et al., 2021; García‐Fernández et al., 2021), and yield‐related attributes (Izquierdo et al., 2023; Mir et al., 2021).

Next to seeds, leaf traits are critical targets for investigation due to their central role in photosynthesis, which directly influences plant productivity and yield potential (Zhu et al., 2010). Leaf morphology, including size, shape, and color, plays a critical role in photosynthetic efficiency, light capture, transpiration, and stress response. As such, these traits are closely linked to yield and resilience under environmental stress, making them valuable targets for genetic improvement, particularly in the context of climate change (Chitwood & Sinha, 2016; Niinemets, 2010). To date, a GWAS on drought tolerance was conducted including leaf elongation rate highlighting a QTL on Pv03 (Hoyos‐Villegas et al., 2017) and a QTL analysis focusing on leaf allometry (Zhang, Zhang, et al., 2020). However, comprehensive GWAS covering diverse leaf morphological traits in *P. vulgaris* are still lacking and none yet have leveraged high‐throughput image phenotyping for leaf shape traits in large panels.

In this study, through the application of image‐based phenotyping, we mapped 71 morphological and colorimetric traits related to leaves and seeds using a panel of 434 *P. vulgaris* accessions genotyped by sequencing (GBS), as well as a subset of 200 accessions sequenced using whole‐genome sequencing (WGS). By integrating both single‐nucleotide polymorphism (SNP) and structural variant (SV) data, we identified 73 high‐confidence marker‐trait associations (MTAs). We provide 114 candidate genes of 25 QTL involved in seed and leaf morphology and pigmentation. Notably, we validate *PvOFP5* as a key determinant of seed size using heterologous expression in *Arabidopsis thaliana*, comparing superior and inferior alleles from *P. vulgaris*. The genetic architecture of leaf and seed morphological traits presented here not only provides insights into domestication and diversification processes but also supports marker‐assisted selection for improved varieties. This is particularly relevant for developing resilient, high‐yielding cultivars in the face of global food demand and environmental challenges. These findings provide new insights into the genetic architecture of the common bean phenome and offer a foundation for future molecular breeding efforts.

## RESULTS

### Analysis of morphological and colorimetric traits in the common bean diversity panel

**Figure 1:**
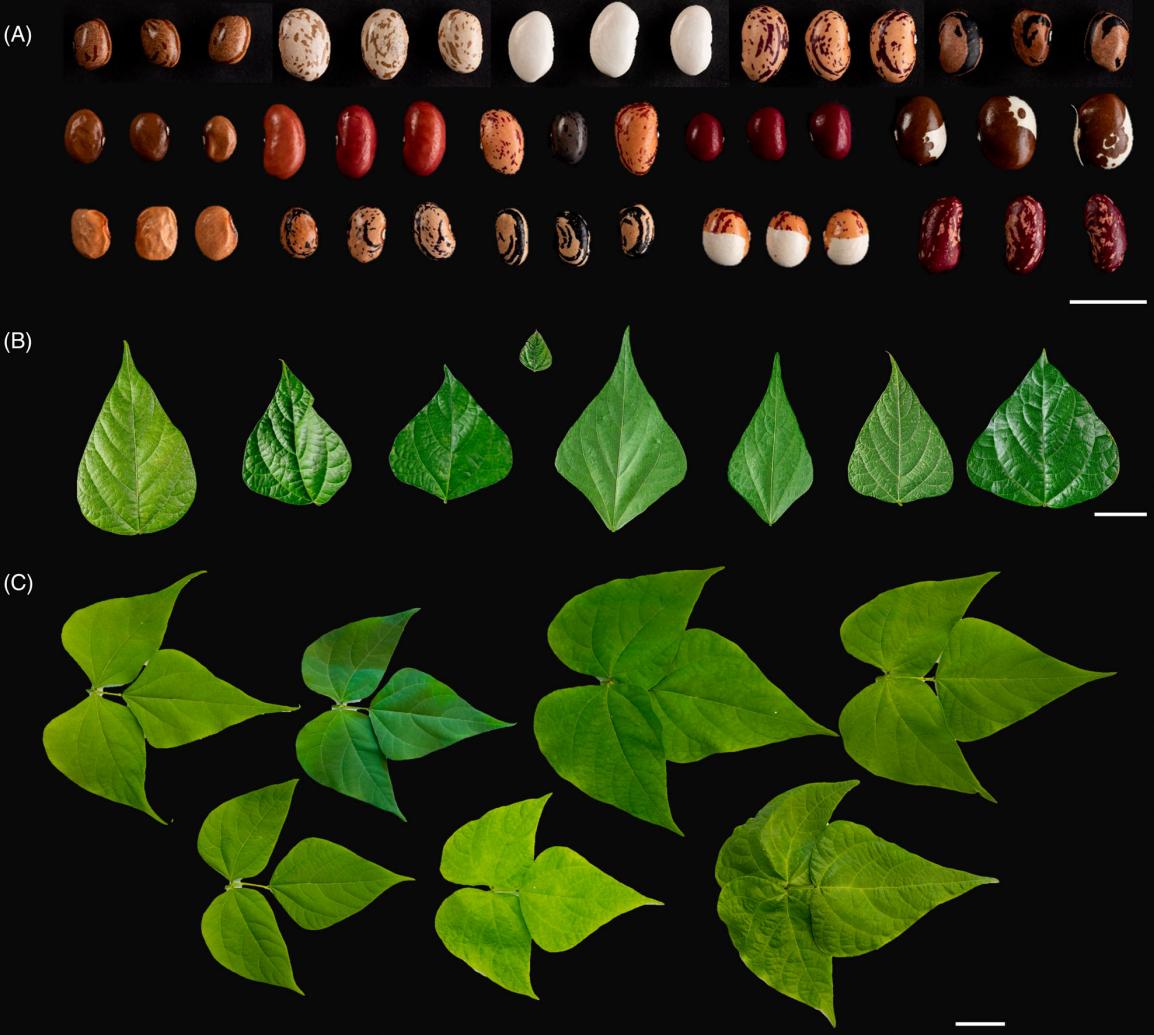
Seeds and leaves of the bean diversity panel. Seed and leaf morphological and colorimetric traits were measured including: weight, area, perimeter, circularity, height, and width, gray value, integrated density, L*, a*, b* values, RGB values, and reflectance across the visible spectrum: violet (380–450 nm), blue (450–495 nm), green (495–570 nm), yellow (570–590 nm), orange (590–620 nm), and red (620–750 nm). Morphological descriptors were obtained by using seed and leaf images. Images of (A) seeds and (B) single leaves were obtained from the full 434 panel and (C) trifoliate leaves were obtained from accessions of the 200 panel. Scale bar = 5 cm.

We analyzed seed and leaf morphological and colorimetric traits of a common bean diversity panel comprising 434 accessions (Figure 1; Figure S1; Table S1) and subjected these traits to GWAS using both GBS data on 434 accessions and WGS‐derived data on a selected subset of 200 accessions. The subset accessions were selected to maximize genetic diversity and ensure balanced representation of the major gene pools identified in the GBS dataset. GBS of the full 434 accession panel yielded 4771 SNPs, whereas WGS of a representative 200‐accession subset identified 3.57 million SNPs and 7100 duplications and deletions, collectively referred to as SVs. Population structure analysis using the GBS data assigned accessions to either the Mesoamerican or Andean gene pools, or as admixed (for accessions with <80% assignment to either pool; Figure S2A). Principal component analysis (PCA) using GBS data revealed that PC1 explained 51.66% of the total genetic variance, clearly separating the two gene pools, while PC2 explained 3.96% of the within‐group variation. Similar patterns were observed using WGS‐derived SNP and SV data: for SNPs, PC1, and PC2 explained 57.19 and 6.06% of the variance, respectively, and for SVs, 40.47 and 4.37% (Figure S2b).

Morphological traits recorded for seeds included weight, area, perimeter, circularity, height, and width (Table 1; Table S2). Colorimetric descriptors encompassed gray value, integrated density, L*, a*, b* values, RGB values, and reflectance across the visible spectrum: violet (380–450 nm), blue (450–495 nm), green (495–570 nm), yellow (570–590 nm), orange (590–620 nm), and red (620–750 nm). In a follow‐up experiment, we rephenotyped the 200 WGS accessions by measuring seed size, L*, a*, b* values, X/Y/Z spectra, and visible light wavelengths.

**Table 1:** Seed and leaf morphological and colorimetric traits

Trait	Tissue_panel	Admixed	AND	MES	CV	Range
Color						
Spectra integral	Leaf_200	388 779.544 ± 12 862.768^ab^	378 090.449 ± 5445.245^a^	408 302.921 ± 8147.892^b^	15.36	288 950.316–619 860.033
520/580	Leaf_200	14 293.19 ± 723.105^a^	13 448.985 ± 294.308^a^	14 994.062 ± 428.332^a^	22.561	8732.433–25 702.44
Chlorophyll	Leaf_200	744.671 ± 20.3^ab^	746.718 ± 8.285^a^	819.424 ± 14.632^b^	13.59	551.684–1244.74
Infrared	Leaf_200	11 741.818 ± 330.698^a^	11 492.945 ± 156.933^a^	11 849.675 ± 186.216^a^	12.669	8381.377–15 664.395
R	Leaf_200	72.128 ± 1.928^ab^	70.601 ± 0.748^a^	75.302 ± 1.125^b^	11.575	56.166–105
G	Leaf_200	94.159 ± 2.187^a^	91.584 ± 0.926^a^	95.86 ± 1.212^a^	10.068	75.333–126
B	Leaf_200	48.44 ± 1.562^a^	49.372 ± 0.383^a^	51.219 ± 0.466^a^	8.193	37.666–60.666
H	Leaf_200	90.211 ± 0.881^a^	91.345 ± 0.444^a^	90.122 ± 0.547^a^	4.704	79.333–101.222
S	Leaf_200	48.184 ± 1.893^a^	45.525 ± 0.712^a^	45.508 ± 0.732^a^	14.052	30–64.555
B.V.	Leaf_200	36.958 ± 0.856^a^	35.927 ± 0.364^a^	37.594 ± 0.474^a^	10.068	29.444–49.333
X	Leaf_200	7.795 ± 0.38^ab^	7.417 ± 0.15^a^	8.411 ± 0.24^b^	22.319	5.027–14.82
Y	Leaf_200	9.924 ± 0.491^ab^	9.385 ± 0.197^a^	10.555 ± 0.301^b^	22.385	6.344–18.336
Z	Leaf_200	3.443 ± 0.141^a^	3.428 ± 0.04^a^	3.736 ± 0.057^b^	12.976	2.388–5.554
L	Leaf_200	37.23 ± 0.856^ab^	36.28 ± 0.357^a^	38.018 ± 0.472^b^	9.895	30.183–49.901
a	Leaf_200	−15.207 ± 0.456^a^	−14.427 ± 0.197^a^	−14.38 ± 0.185^a^	−11.721	−18.916 to −10.094
b	Leaf_200	23.03 ± 1.276^a^	21.337 ± 0.51^a^	22.424 ± 0.611^a^	22.349	11.97–36.247
Violet	Leaf_200	3.946 ± 0.156^ab^	3.981 ± 0.044^a^	4.217 ± 0.056^b^	11.652	2.844–5.893
Blue	Leaf_200	4.012 ± 0.175^ab^	3.993 ± 0.048^a^	4.388 ± 0.07^b^	13.624	2.756–6.644
Green	Leaf_200	10.483 ± 0.499^ab^	9.915 ± 0.202^a^	11.062 ± 0.298^b^	21.296	6.689–18.728
Yellow	Leaf_200	9.803 ± 0.526^ab^	9.246 ± 0.211^a^	10.52 ± 0.33^b^	24.59	6.135–19.095
Orange	Leaf_200	6.848 ± 0.371^ab^	6.518 ± 0.144^a^	7.586 ± 0.247^b^	25.299	4.151–14.115
Red	Leaf_200	20.704 ± 0.566^a^	20.336 ± 0.267^a^	21.305 ± 0.353^a^	12.942	15.299–28.978
X	Seed_200	18.984 ± 4.46^a^	20.405 ± 1.388^a^	28.413 ± 2.649^a^	77.408	0.667–69.794
Y	Seed_200	18.364 ± 4.628^a^	19.174 ± 1.442^a^	28.222 ± 2.765^a^	83.503	0.673–72.338
Z	Seed_200	9.373 ± 2.767^a^	9.6 ± 0.902^a^	17.633 ± 1.993^a^	103.4	0.57–52.392
L	Seed_200	41.354 ± 5.995^a^	46.361 ± 1.916^a^	50.984 ± 3.142^a^	47.062	5.844–88.001
a	Seed_200	9.322 ± 2.013^ab^	11.941 ± 0.872^a^	6.777 ± 0.992^b^	89.254	−1.555 to 33.997
b	Seed_200	15.658 ± 3.497^ab^	18.092 ± 1.4^a^	12.081 ± 1.221^b^	79.206	−8.191 to 54.91
Violet	Seed_200	8.781 ± 2.809^a^	9.391 ± 0.972^a^	18.705 ± 2.189^a^	110.738	0.707–57.855
Blue	Seed_200	12.598 ± 3.696^a^	12.623 ± 1.168^a^	22.631 ± 2.538^a^	101.735	0.656–66.32
Green	Seed_200	16.187 ± 4.421^a^	16.381 ± 1.385^a^	26.088 ± 2.725^a^	91.777	0.659–71
Yellow	Seed_200	19.402 ± 4.883^a^	20.184 ± 1.535^a^	29.321 ± 2.84^a^	82.761	0.674–73.526
Orange	Seed_200	21.894 ± 5.015^a^	23.892 ± 1.566^a^	31.725 ± 2.851^a^	73.785	0.688–74.53
Red	Seed_200	31.641 ± 5.179^a^	36.497 ± 1.518^a^	39.326 ± 2.919^a^	53.471	0.754–77.635
Gray value minimum	Seed_434	11.88 ± 1.958^ab^	7.711 ± 0.453^a^	12.998 ± 0.977^b^	100.799	0–37
Gray value maximum	Seed_434	226.16 ± 2.759^a^	221.808 ± 0.92^a^	222.13 ± 1.768^a^	8.029	152.666–250.285
Gray value mean	Seed_434	99.861 ± 10.182^a^	86.242 ± 2.79^a^	104.474 ± 5.266^a^	58.74	16.766–207.295
Gray value median	Seed_434	91.698 ± 10.905^a^	77.804 ± 3.092^a^	98.52 ± 5.566^a^	68.726	5.1–208.857
Integrated density	Seed_434	4 993 207.08 ± 540 372.355^a^	5 228 120.303 ± 201 068.03^a^	5 823 717.804 ± 398 774.579^a^	72.617	346 741.456–20 827 653.246
L	Seed_434	51.916 ± 3.65^a^	48.515 ± 1.077^a^	52.967 ± 1.878^a^	39.942	9.749–88.004
a	Seed_434	7.151 ± 0.981^a^	13.211 ± 0.425^b^	7.977 ± 0.605^a^	69.267	0–30.765
b	Seed_434	15.561 ± 1.648^a^	19.757 ± 0.689^b^	14.221 ± 0.807^a^	61.829	0–51.556
R	Seed_434	0.517 ± 0.036^a^	0.51 ± 0.01^a^	0.529 ± 0.018^a^	39.031	0.077–0.866
G	Seed_434	0.422 ± 0.036^a^	0.358 ± 0.01^a^	0.43 ± 0.019^a^	52.768	0.072–0.832
B	Seed_434	0.337 ± 0.033^ab^	0.268 ± 0.008^a^	0.356 ± 0.017^b^	59.896	0.052–0.752
Violet	Seed_434	0.145 ± 0.02^a^	0.1 ± 0.005^a^	0.158 ± 0.011^a^	89.29	0.01–0.425
Blue	Seed_434	0.2 ± 0.03^a^	0.126 ± 0.007^a^	0.218 ± 0.016^a^	101.39	0.012–0.629
Green	Seed_434	0.249 ± 0.034^a^	0.17 ± 0.008^a^	0.261 ± 0.018^a^	89.673	0.01–0.703
Yellow	Seed_434	0.31 ± 0.036^a^	0.248 ± 0.01^a^	0.318 ± 0.018^a^	71.468	0.011–0.74
Orange	Seed_434	0.323 ± 0.035^a^	0.28 ± 0.009^a^	0.337 ± 0.018^a^	63.184	0.011–0.751
Red	Seed_434	0.313 ± 0.034^a^	0.273 ± 0.009^a^	0.33 ± 0.017^a^	62.687	0.01–0.745
Morphology						
Extent	Leaf_200	0.505 ± 0.01^a^	0.505 ± 0.004^a^	0.504 ± 0.004^a^	8.087	0.413–0.614
Solidity	Leaf_200	0.718 ± 0.013^a^	0.711 ± 0.003^a^	0.753 ± 0.004^b^	5.656	0.62–0.833
Eccentricity	Leaf_200	0.452 ± 0.018^ab^	0.454 ± 0.009^a^	0.424 ± 0.008^b^	18.573	0.173–0.625
Centroid 1	Leaf_200	1512.238 ± 13.228^a^	1514.131 ± 6.722^a^	1562.552 ± 5.751^b^	3.88	1370.478–1719.057
Centroid 2	Leaf_200	1403.754 ± 50.446^b^	1461.623 ± 18.875^b^	1297.103 ± 14.003^a^	12.333	1059.591–1871.124
Roundness	Leaf_200	0.339 ± 0.014^ab^	0.32 ± 0.004^a^	0.346 ± 0.004^b^	13.45	0.201–0.447
Area [cm^2^]	Leaf_200	373.304 ± 28.334^ab^	415.926 ± 11.659^a^	322.653 ± 8.552^b^	8.552	27.527–162.195
Perimeter [cm]	Leaf_200	117.508 ± 5.437^a^	127.473 ± 2.011^b^	107.883 ± 1.56^a^	1.56	15.979–81.513
Extent	Leaf_434	0.569 ± 0.005^a^	0.564 ± 0.002^a^	0.585 ± 0.002^b^	5.055	0.475–0.66
Solidity	Leaf_434	0.938 ± 0.004^ab^	0.932 ± 0.002^a^	0.948 ± 0.001^b^	2.181	0.854–0.991
Roundness	Leaf_434	0.629 ± 0.009^a^	0.613 ± 0.004^a^	0.651 ± 0.005^b^	8.496	0.4–0.754
Area [cm^2^]	Leaf_434	87.279 ± 5.501^a^	97.533 ± 2.402^a^	95.687 ± 1.999^a^	1.999	24.127–43.487
Perimeter [cm]	Leaf_434	41.129 ± 1.239^a^	44.238 ± 0.561^a^	42.937 ± 0.571^a^	0.571	13.476–25.937
Size [mm^2^]	Seed_200	66.002 ± 3.176^a^	84.291 ± 1.985^b^	66.611 ± 3.086^a^	31.02	30.102–137.92
Circularity	Seed_434	0.665 ± 0.005^a^	0.655 ± 0.002^a^	0.695 ± 0.002^b^	6.424	0.521–0.803
Weight [g]	Seed_434	1.015 ± 0.039^a^	1.35 ± 0.02^b^	0.947 ± 0.028^a^	32.566	0.405–2.438
Area [mm^2^]	Seed_434	16.448 ± 0.594^a^	19.895 ± 0.282^a^	16.36 ± 0.515^b^	0.515	5.916–29.899
Perimeter [mm]	Seed_434	18.796 ± 0.415^a^	21.002 ± 0.183^a^	18.114 ± 0.33^b^	0.33	10.193–18.371
Height [mm]	Seed_434	5.182 ± 0.107^a^	5.718 ± 0.046^a^	4.897 ± 0.079^b^	0.079	3.28–17.017
Width [mm]	Seed_434	4.472 ± 0.127^a^	4.958 ± 0.049^a^	4.501 ± 0.082^b^	0.082	2.285–19.049

*Note*: Data are shown as mean ± standard error. Letters indicate significance (*P*‐adj <0.05) between Andean (AND), Mesoamerican (MES) gene pool, and admixed calculated with either Kruskal–Wallis *post‐hoc* Dunn‐test or anova *post‐hoc* Tukey HSD test as indicated based on the data normal distribution.

Abbreviation: CV, coefficient of variation.

In addition to seed traits, we characterized apical leaflet morphology in all 434 accessions, assessing extent, solidity, area, perimeter, and roundness. For the 200‐accession subset, we extended this analysis to the trifoliate leaves, capturing morphological traits (area, extent, solidity, perimeter, eccentricity, centroid, and roundness) and colorimetric descriptors (spectral integral, chlorophyll content, infrared reflectance, R/G/B, H/S/V, X/Y/Z, L*/a*/b*, and reflectance from violet to red; Figure 1B,C).

We then assessed whether morphological and colorimetric traits differed significantly between the Mesoamerican and the Andean gene pools. In the trifoliate leaves, Mesoamerican accessions exhibited significantly higher absorbance in the violet, blue, green, yellow, orange, and red wavelengths, as well as for L*, spectral integral, chlorophyll, and X/Y/Z color metrics (Table 1; Table S2; Figure S3). Morphologically, Mesoamerican trifoliate leaves showed higher solidity, roundness, and centroid 1 value, but lower perimeter, area, and centroid 2 values compared to Andean accessions (Table 1; Table S2; Figure S4). For the apical leaflets, solidity, roundness, and extent were also significantly higher in the Mesoamerican gene pool; however, unlike the trifoliate leaves, leaf area did not differ significantly between pools.

In the 200‐accession panel, Mesoamerican seeds had significantly smaller seed size and lower a* and b* color values (Table 1; Table S2; Figure S5). For the full 434‐panel, Mesoamerican seeds exhibited higher minimum gray value, blue spectrum reflectance, and circularity, while a*, b*, area, weight, height, width, and perimeter were all significantly lower (Table 1; Table S2; Figure S6).

In summary, Mesoamerican plants tend to have leaves with higher color intensity and more compact, rounded shapes, while Andean plants have larger, less compact leaves. Mesoamerican seeds are generally smaller, lighter in color, and more circular, whereas Andean seeds are larger, more elongated, and more intensely colored. These differences reflect distinct morphological and colorimetric traits between the two gene pools.

**Figure 2:**
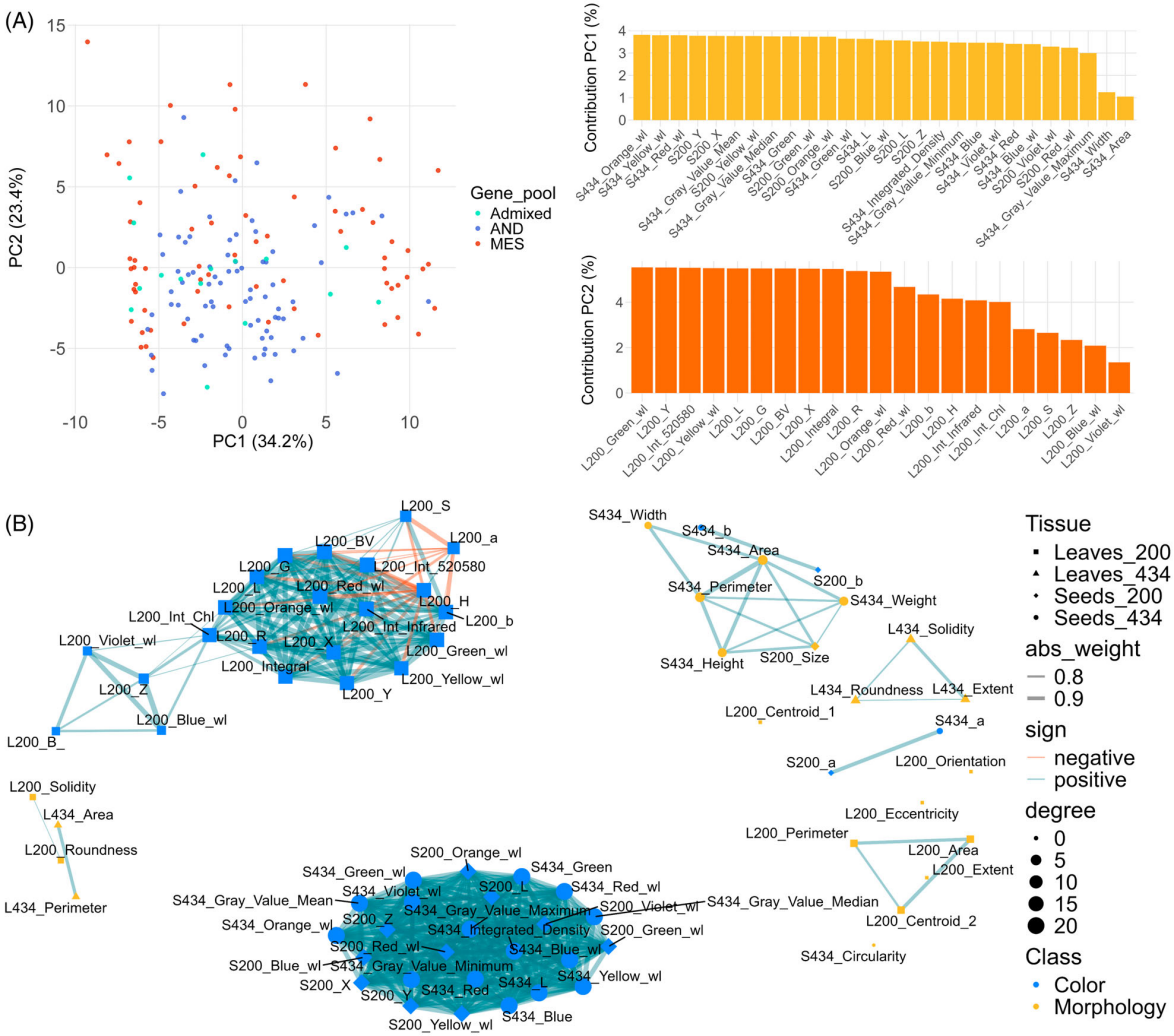
Colorimetric and morphological descriptors of leaves and seeds. (A) Principal component analysis (PCA) of Andean (AND), Mesoamerican (MES), and admixed common bean accessions including variable contribution to PC1 and PC2. PC1 explains the separation of accessions mainly by seed descriptors while PC2 by leaf descriptors. (B) Network analysis using |*r*| ≥0.7 and *P* < 0.05. L/S200 = Leaf and seed descriptors from the 200 panel, L/S434 = Leaf and seed descriptors from the 434 panel.

PCA of leaf and seed morphological and colorimetric traits revealed that PC1 and PC2 accounted for 34.2 and 23.4% of the total variance, respectively (Figure 2A). Interestingly, no clear separation was observed between Andean and Mesoamerican accessions based on phenotypic and colorimetric traits. Analysis of variable contributions indicated that variation along PC1 was primarily driven by seed colorimetric traits, while leaf colorimetric traits contributed most to the variation along PC2. We performed a correlation network analysis and identified 10 clusters comprising ≥2 features, of which five were color‐related and five morphology‐related (Figure 2B). Of the top three clusters, two were color‐related comprising 25 seed color features and 18 leaf color features, and one morphology‐related cluster comprising six seed morphology features (size, area, height, perimeter, weight, width; Table S3). These phenotypic differences between gene pools reflect domestication‐related divergence and lay the foundation for GWAS to dissect the genetic architecture of color and morphology in *P. vulgaris*.

### The genetic architecture of the common bean leaf and seed phenome

We performed GWAS for a total of 71 morphological and colorimetric descriptors across the common bean diversity panel. Using GBS data, we identified 35 high‐confidence MTAs, defined as associations detected by at least two of the five GWAS models (CMLM, MLM, MLMM, BLINK, and FarmCPU). Similarly, WGS identified 24 MTAs based on SVs and 15 MTAs based on SNPs (Figure S7; Tables S4–S7). In total, we identified four QTL previously reported in common bean GWAS analyses (García‐Fernández et al., 2021; Hagerty et al., 2016; Murube et al., 2020). For instance, a MYB transcription factor (*Phvul.004G144900*) associated in our dataset with multiple seed color traits—orange, red, yellow, gray value, green, and L*—is also linked to pod width, height, and wall thickness in earlier studies (García‐Fernández et al., 2021; Hagerty et al., 2016). Moreover, two QTL located on chromosome 11 were consistent with prior findings (García‐Fernández et al., 2021; Murube et al., 2020). The first QTL, located at 2.3 Mb and associated with violet and X seed color, encompassed two candidate genes: a basic helix–loop–helix (bHLH) transcription factor (*Phvul.011G026500*) and a WD40 repeat protein (*Phvul.011G028400*), which share 17 and 19% sequence similarity, respectively, with the WD40 proteins ZmKRN2 and OsKRN2 known to regulate grain yield in maize and rice (Chen et al., 2022). The second QTL, located at 4.2 Mb, was linked to blue, green, and violet seed wavelengths and included a bZIP transcription factor (*Phvul.011G047100*) and a probable lipid transfer protein (*Phvul.011G047400*).

Across all datasets, we detected 21 novel QTL and 114 candidate genes. Of these, five QTL were identified from GBS data, eight from WGS‐SV data, and one from WGS‐SNP data using at least two GWAS models, corresponding to 10, 51, and 3 candidate genes, respectively (Figure S8; Table S8). In addition, we identified 32, 10, and 7 additional QTL from GBS, WGS‐SV, and WGS‐SNP datasets, respectively.

**Figure 3:**
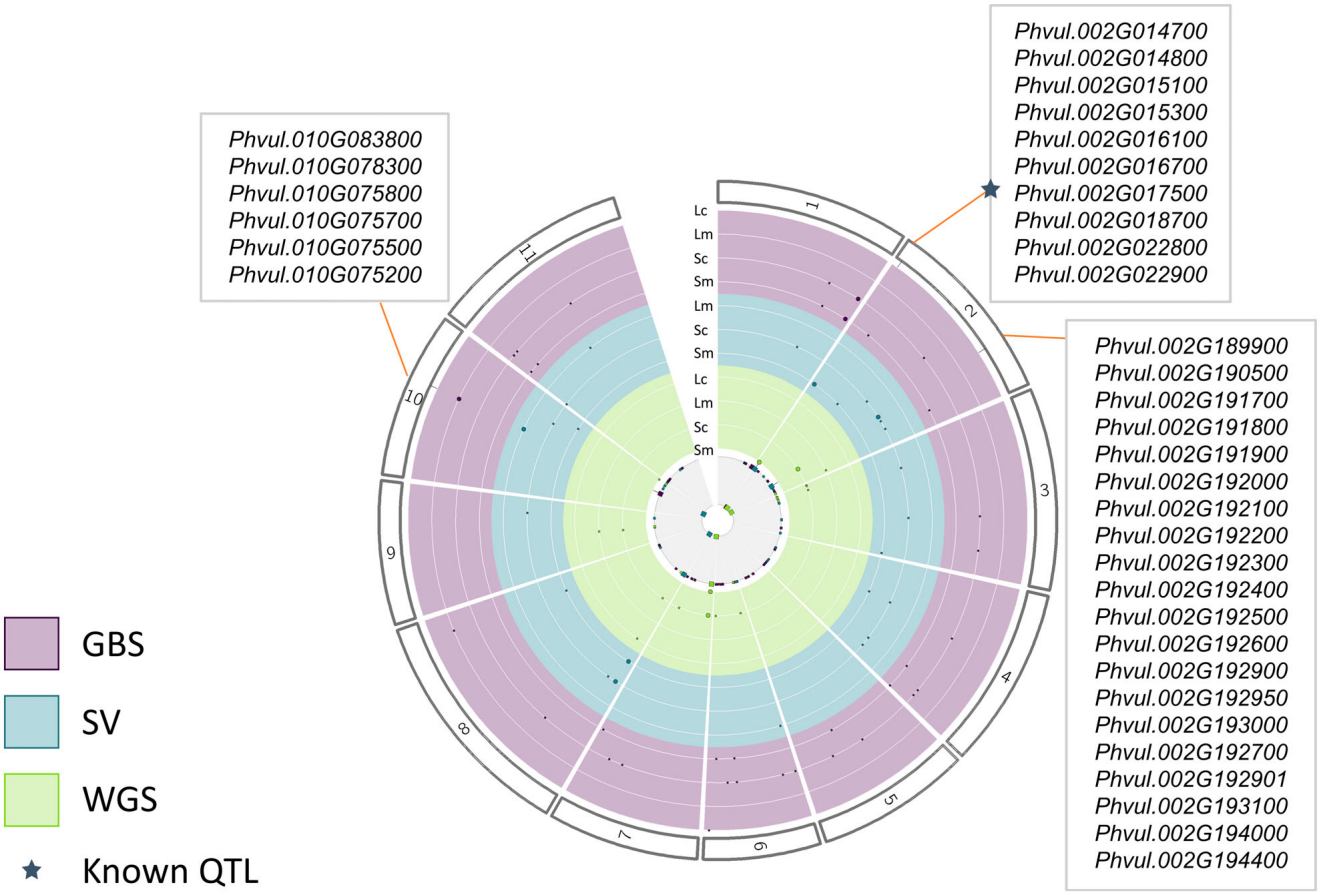
Common bean phenotype QTLome using genotype‐by‐sequencing (GBS), structural variance (SV) and whole‐genome sequencing (WGS). Counts of associated traits of leaf and seed color and morphology using WGS, SV and GBS data. Only significant single‐nucleotide polymorphisms (SNPs) identified in ≥ two GWAS methods were considered significant. Common QTL across all panels are highlighted with a candidate gene. Lc, leaf color traits; Lm, leaf morphology traits; QTL, quantitative trait locus; Sc, seed color traits; Sm, seed morphology traits.

To assess pleiotropic effects across the three datasets, we compared significant signals (≥2 GWAS models) and identified three shared QTL comprising 35 candidate genes (Figure 3). Two pleiotropic QTL were located on chromosome 2 and were detected in both WGS‐SNP and WGS‐SV analyses. The first, located at 1.77 Mb, contained eight candidate genes associated with seed perimeter, area, and width and was previously identified by García‐Fernández et al. (2021) to be associated with pod perimeter and WRKY transcription factor (*Phvul.002G016100*), MYB transcription factor (*Phvul.002G015100*) and two CYP82C2‐related genes (*Phvul.002G022800*, *Phvul.002G022900*) as candidate genes. Here, we present six more candidates: These included two isoflavone 2′‐monooxygenases (*Phvul.002G014700*, *Phvul.002G014800*), a microtubule‐associated protein (*Phvul.002G015300*), an ethylene‐responsive transcription factor (*Phvul.002G016700*), a caffeoyl‐CoA *O*‐methyltransferase (*Phvul.002G017500*), and a zeaxanthin epoxidase (*Phvul.002G018700*). Expression data support the functional relevance of these candidates, with *Phvul.002G014800* expressed in Andean domesticated pods, high transcript levels of *Phvul.002G014700*, *Phvul.002G016700*, and *Phvul.002G018700* at 40 days post‐anthesis (DPA), and mature black seeds exhibiting elevated expression of *Phvul.002G015100*, *Phvul.002G015300*, *Phvul.002G016100*, and *Phvul.002G017500* (Parreira et al., 2018; Perez de Souza et al., 2019; Roy et al., 2025).

The second QTL on chromosome 2, located at 35.56 Mb, was associated with seed gray value and comprised 20 candidate genes. These included a VQ‐motif‐containing protein (*Phvul.002G189900*), a PLATZ transcription factor (*Phvul.002G190500*), eight CCR4‐NOT transcription complex subunits (*Phvul.002G191700*, *Phvul.002G191800*, *Phvul.002G191900*, *Phvul.002G192100*, *Phvul.002G192300*, *Phvul.002G192400*, *Phvul.002G192500*, *Phvul.002G192900*), five CCR4‐associated factor 1 homolog 11‐related genes (*Phvul.002G192200*, *Phvul.002G192950*, *Phvul.002G193000*, *Phvul.002G192700*, *Phvul.002G193100*), three glycerol‐3‐phosphate *O*‐acyltransferases (*Phvul.002G192000*, *Phvul.002G192600*, *Phvul.002G192901*), a flavin reductase‐related gene (*Phvul.002G194000*), and a bHLH transcription factor (*Phvul.002G194400*). Transcriptomic data support their developmental relevance: high expression was observed in Andean flowers (*Phvul.002G189900*, *Phvul.002G192000*), Mesoamerican wild pods (*Phvul.002G192600*, *Phvul.002G194000*, *Phvul.002G194400*), 10 DPA (*Phvul.002G190500*), mature black seeds (*Phvul.002G191700*, *Phvul.002G191800*, *Phvul.002G191900*, *Phvul.002G193000*), and in final seed developmental stages (*Phvul.002G192000*, *Phvul.002G191900*, *Phvul.002G193000*) (Jurado et al., 2024; Parreira et al., 2018; Perez de Souza et al., 2019; Roy et al., 2025).

A third pleiotropic QTL was located on chromosome 10 (26.75 Mb) and was identified in both GBS and WGS‐SV analyses. This region was associated with trifoliate leaf roundness and perimeter and comprised six candidate genes, including a MYB/SANT‐like DNA‐binding protein (*Phvul.010G083800*), a MYB domain protein 100‐related gene (*Phvul.010G078300*), two trichome birefringence‐like 3 genes (*Phvul.010G075700*, *Phvul.010G075800*) moderately expressed in Mesoamerican wild pods (Perez de Souza et al., 2019), an AtMYB1‐like gene (*Phvul.010G075500*) expressed at 40 DPA and in mature seeds (Jurado et al., 2024; Parreira et al., 2018), a pectinacetylesterase family protein (*Phvul.010G075200*), an ABC transporter G family member 38 (*Phvul.010G076901*), highly expressed in Andean domesticated flowers (Perez de Souza et al., 2019), a CYP71B21‐related gene (*Phvul.010G079100*) expressed in Andean domesticated flowers, at 40 DPA, and in mature seeds (Jurado et al., 2024; Parreira et al., 2018; Perez de Souza et al., 2019), and a MATE efflux family protein (*Phvul.010G068200*).

Additionally, a GBS‐derived QTL on chromosome 6 was associated with multiple colorimetric traits, including the green, orange, and yellow wavelengths; X and H color coordinates; the 520/580 nm wavelength ratio; and R and Y components. Candidate genes in this region included a flavonol 3‐*O*‐glucosyltransferase (*Phvul.006G201100*) highly expressed in Mesoamerican wild pods, Andean domesticated flowers and pods, and 10 DPA (Parreira et al., 2018; Perez de Souza et al., 2019), an SRF‐type transcription factor (*Phvul.006G202300*) highly expressed in black seeds (Roy et al., 2025), and a SAC51‐related transcription factor (*Phvul.006G203400*) highly expressed in black seeds, 40 DPA, and in mature seeds (Jurado et al., 2024; Parreira et al., 2018; Roy et al., 2025).

In total, we identified 66 candidate genes associated with seed color, 20 with seed morphology, 14 with both seed color and morphology, 19 with leaf morphology, and 3 with leaf color. Among these, we found 59 transcription factors, 14 monooxygenases, 14 transferases, and 6 transporters, highlighting the diverse molecular mechanisms underlying variation in seed and leaf traits in common bean.

### Functional validation of PvOFP5 as a key player regulating the seed size in common bean

**Figure 4:**
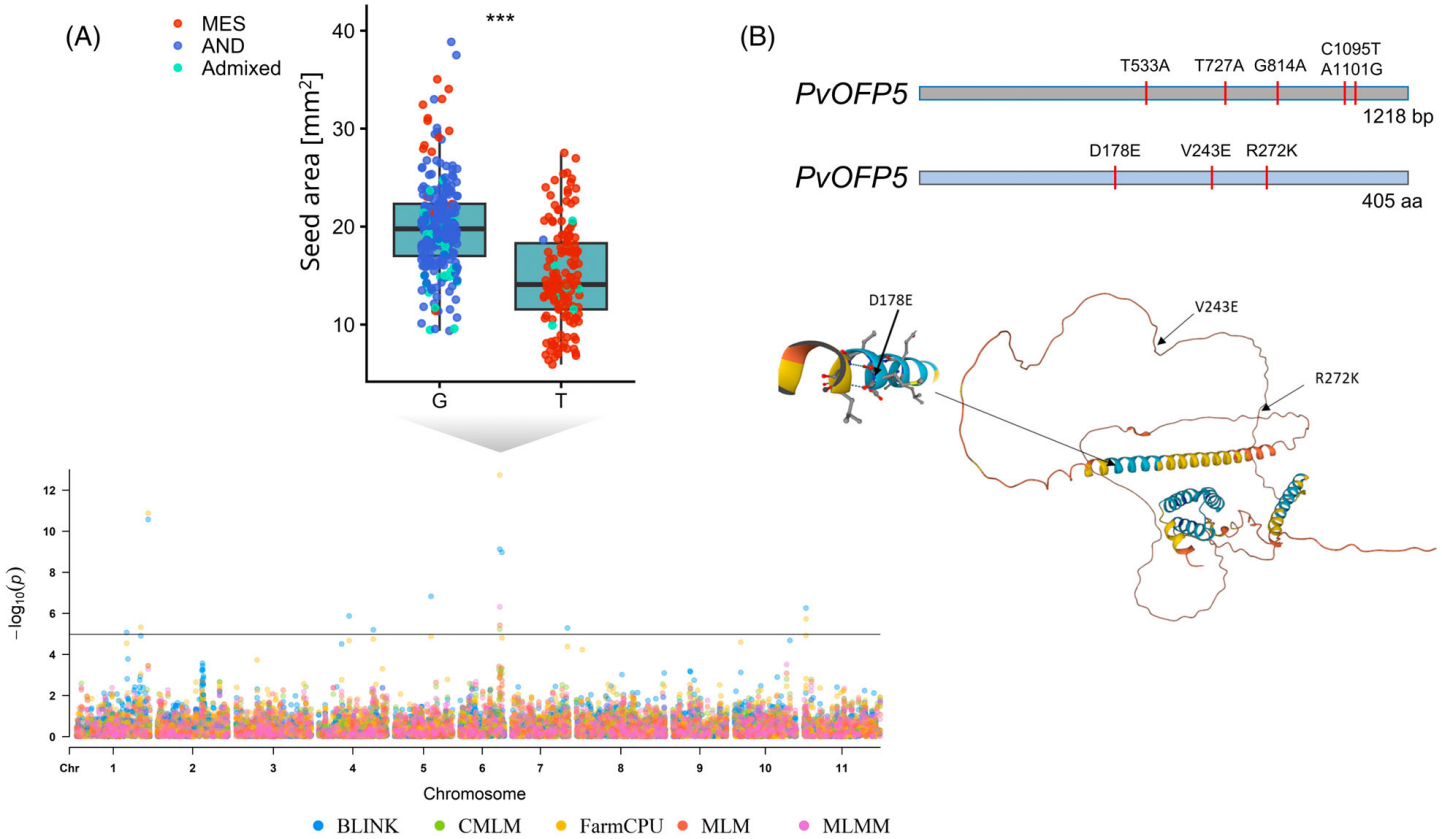
Identification of PvOFP5 as seed size determinant. (A) Manhattan plot overlay of BLINK, CMLM, FarmCPU, MLM, and MLMM of seed area using genotype‐by‐sequencing (GBS) data and haplotype plot of seed area in mm^2^ with dots representing the accession's gene pool. Chromosome 6 shows an association with a quantitative trait locus (QTL) comprising *PvOFP5*. (B) Cloning of *PvOFP5* identified five single‐nucleotide polymorphisms (SNPs) at indicated positions in Ece122 with low seed area, a Mesoamerican gene pool accession, compared to Ece153 with high seed area, an Andean gene pool accession, translating to three amino acid substitutions. D178E is located in an alpha‐helix. Asterisks indicate significant differences as assessed by Student’s *t*‐test (*P* < 0.001).

Seed area is a major determinant of yield in common bean. In this study, we identified a prominent QTL located on chromosome 6. This locus was detected in the GBS dataset but not in the WGS dataset, potentially due to differences in population size and sequencing approach. This QTL harbors the candidate gene *PvOFP5*, a 1218 bp gene encoding an ovate family protein, located at position 27.49 Mb (Figure 4; Table S9). Using WGS‐SNP data, the *r* ^2^ between the target SNP (S06_27905417) and the nearest gene SNP (S06_27485455) was 0.497, indicating moderate linkage disequilibrium. We cloned two alleles of *PvOFP5*: the inferior allele (OFP5‐1) from the Mesoamerican gene pool and the superior allele (OFP5‐2) from the Andean gene pool. Comparative sequence analysis revealed five SNPs (T533A, T727A, G814A, C1095T, A1101G) comparing Ece122 (MES) to Ece153 (AND) that result in three amino acid substitutions: D178E, V243E, and R272K. Structural predictions using AlphaFold suggested that the D178E substitution occurs within an alpha‐helix, while the V243E and R272K changes are located in unstructured regions.

Due to the challenges associated with stable transformation and long generation time in common bean, we used *A. thaliana* as a heterologous system to investigate the functional role of these alleles. Both *PvOFP5* alleles were overexpressed in the Columbia‐0 (Col‐0) wild‐type (WT) background and used to complement the *ofp5* T‐DNA knockout (KO) line. For the WT overexpression (OX) lines, we obtained two independent biological replicates per allele, while three and five independent lines were obtained for the OFP5‐1 and OFP5‐2 complementation lines, respectively.

**Figure 5:**
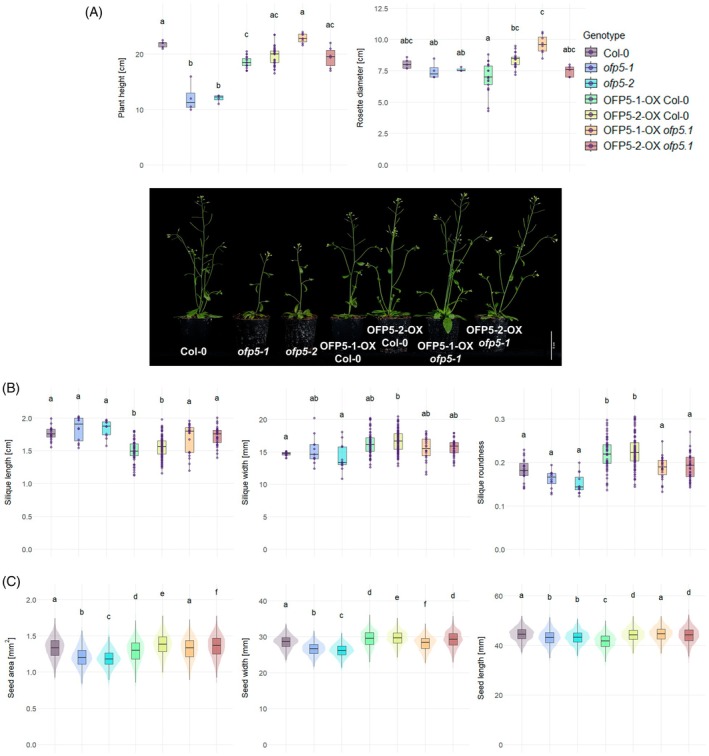
Both bean alleles *PvOFP5* complement the *Arabidopsis thaliana* AtOFP5 knockout lines. The Mesoamerican OFP5‐1 allele (five SNPs) as well as the Andean OFP5‐2 (no SNPs) complement the *A. thaliana* knockout line ofp5‐1 (two independent biological replicates) and the overexpression (OX) in wild type Columbia‐0 (Col‐0; three and five biological independent replicates for the OFP5‐1 and OFP5‐2 alleles) shows a slightly reduced height. (A) Height and rosette diameter of 4‐week‐old plants (*n*_Col‐0_ = 5, *n*_ofp5‐1_ = 4, *n*_ofp5‐2_ = 4, *n*_OFP5‐1‐OX Col‐0_ = 21, *n*_OFP5‐2‐OX Col‐0_ = 33, *n*_OFP5‐1‐OX ofp5‐1_ = 8, *n*_OFP5‐2‐OX ofp5‐1_ = 6) and phenotypic image with respective mutant lines. (B) Length [cm], width [mm] and roundness of the siliques of 5‐week‐old plants (*n*_Col‐0_ = 19, *n*_ofp5‐1_ = 12, *n*_ofp5‐2_ = 12, *n*_OFP5‐1‐OX Col‐0_ = 63, *n*_OFP5‐2‐OX Col‐0_ = 100, *n*_OFP5‐1‐OX ofp5‐1_ = 23, *n*_OFP5‐2‐OX ofp5‐1_ = 34). (C) Seed area [mm^2^], width and length [mm] of wild type, knockout, overexpression, and complementation lines (*n*_Col‐0_ = 4379, *n*_ofp5‐1_ = 2308, *n*_ofp5‐2_ = 2091, *n*_OFP5‐1‐OX Col‐0_ = 3799, *n*_OFP5‐2‐OX Col‐0_ = 7994, *n*_OFP5‐1‐OX ofp5‐1_ = 1516, *n*_OFP5‐2‐OX ofp5‐1_ = 3152). Letters indicate significances *P* < 0.05 calculated either with two‐way anova or Kruskal–Wallis test with *post‐hoc* Tukey's HSD or Dunn‐test, respectively, based on the data normal distribution. SNP, single‐nucleotide polymorphism.

Phenotypic analysis showed that plant height was significantly reduced in both independent *ofp5* KO lines (Figure 5A). Complementation with either allele restored the WT phenotype, but OX of the inferior OFP5‐1 allele resulted in a significantly smaller plant height compared to both WT and the OFP5‐2 OX lines. Rosette diameter was not significantly altered in the KO or complementation lines. However, OX of OFP5‐1 in the WT background led to a reduced rosette diameter compared to the corresponding complementation line. Silique morphology, quantified through image analysis, revealed that WT OX of both alleles led to shorter and rounder siliques relative to WT (Figure 5B). Silique width was significantly increased only in OFP5‐2 OX lines compared to both WT and *ofp5* mutants.

To validate the function of *PvOFP5* in regulating seed size, we used seed cross‐sectional area as a proxy and quantified seed area, length, and width using image‐based phenotyping (Figure 5C). All three traits were reduced in the KO lines. Across all parameters, OFP5‐2 consistently produced larger seeds than OFP5‐1. The inferior allele complemented the seed area and length phenotype in the KO background but led to reduced seed size when overexpressed in WT. In contrast, OX of the superior allele OFP5‐2 led to increased seed area and length compared to WT. Interestingly, seed width showed a more complex pattern: OX of OFP5‐1 in WT resulted in an increase in seed width, while its complementation line showed reduced width. Both OX and complementation of OFP5‐2 led to increased seed width.

Together, these results demonstrate that PvOFP5 plays a key role in regulating seed size in common bean. The superior allele from the Andean gene pool enhances seed area, length, and width, whereas the Mesoamerican allele is associated with smaller seeds. This functional divergence reflects natural allelic variation contributing to this yield‐related trait of seed volume in domesticated common bean.

## DISCUSSION

Seed color and morphology are key traits influencing both consumer preference and breeder selection. In this study, we mapped multiple seed morphological and colorimetric descriptors (Figures 1 and 2; Table 1; Table S2) and conducted GWAS of leaf morphological and colorimetric traits (Figure 3; Tables S4–S7). In addition to SNP‐based mapping, we incorporated SV data to uncover novel loci, particularly those involved in seed morphology. While SNP‐based GWAS have identified numerous trait‐associated loci, SVs—such as insertions and deletions—are increasingly recognized for their capacity to drive substantial phenotypic variation (Alonge et al., 2020). Integrating SVs with SNP data enhances both the resolution and power of association analyses, thereby providing a more comprehensive understanding of the genetic architecture underlying complex traits.

Previous studies (García‐Fernández et al., 2021; Hagerty et al., 2016; Murube et al., 2020) have identified QTL associated with seed morphology and pod color. Interestingly, several co‐localized QTL were reported to influence both seed morphology and color, suggesting potential pleiotropic effects.

In the present analysis, we applied a stringent filtering criterion by considering only SNPs detected in at least two GWAS models (MLM, MLMM, CMLM, FarmCPU, and BLINK). This conservative approach was chosen to minimize false positives arising from multiple testing across numerous traits. While this likely reduced the total number of detected loci, it increased the reliability of identified associations (Table S8). It is plausible that additional candidate genes and QTL could be revealed under a less stringent threshold, particularly for complex polygenic traits.

Despite the well‐established genetic differentiation between Andean and Mesoamerican gene pools, PCA of seed and leaf descriptors revealed no clear separation between these groups (Figure 2A). This lack of phenotypic stratification may suggest convergent selection on these traits, extensive gene flow between the populations, or a high degree of plasticity in the morphological and colorimetric traits analyzed. However, pairwise comparisons did reveal significant differences between Andean and Mesoamerican accessions for many traits (Table 1).

Most traits exhibited positive correlations, reflecting shared developmental or biochemical pathways influencing morphology and pigmentation (Figure 2B). However, some leaf colorimetric parameters, such as leaf saturation and the a* and b* components, showed negative correlations. This may result from opposing pigment contributions (e.g., chlorophyll versus carotenoids) or from light reflectance differences linked to developmental or physiological variation among accessions.

Together, these findings highlight the complex yet interconnected genetic basis of seed and leaf morphology and coloration in common bean. The integration of SNP‐ and SV‐based GWAS approaches revealed both known and novel loci (Figure 3; Table S8), underscoring the importance of combining complementary genomic resources to capture the full spectrum of genetic variation underlying these traits. This comprehensive framework not only refines our understanding of the phenotypic diversity observed across gene pools but also provides a robust foundation for functional validation of key candidate genes, such as *PvOFP5*, implicated in seed size regulation (Figure 4).

### PvOFP5 regulates seed size

Similarly, Alves et al. (2024) mapped seed weight to position 22 116 966 bp on chromosome 6, approximately 5.37 Mb upstream of *PvOFP5*; however, the underlying candidate gene was not reported (Alves et al., 2024). Given that *A. thaliana* is a well‐established heterologous system for functional validation of crop and wild plant genes (Cheng et al., 2020; Niu et al., 2025; Qin et al., 2025), we employed the Mesoamerican (inferior) and Andean (superior) *PvOFP5* alleles to complement the Arabidopsis *ofp5* KO phenotype and to overexpress them in a WT background (Figure 5). Members of the OFP group are known to interact with TONNEAU1 RECRUITMENT MOTIF (TRM) proteins, thereby modulating cell division and determining fruit and organ shape in diverse crops such as tomato, pepper, cucumber, melon, and potato (Snouffer et al., 2020; Tsaballa et al., 2011, 2013; Wang et al., 2016, 2025; Wu et al., 2018). OFPs have also been implicated in drought resilience in rice (Ma et al., 2017) and fruit ripening in banana (Liu et al., 2015). In Arabidopsis, AtOFP5 is essential for female gametophyte and embryo sac development (Pagnussat et al., 2007), and accordingly, the *ofp5* KO exhibits reduced seed size. Remarkably, despite fundamental differences in seed storage composition between Arabidopsis (predominantly lipids) and common bean (proteins and starch), both common bean alleles successfully complemented the KO phenotype. These observations support the notion that seed size regulation may rely, at least in part, on conserved mechanisms among dicotyledonous species. Moreover, the alleles preserved their relative phenotypic effects, with the superior allele conferring larger seeds than the inferior one. These findings highlight the robustness of *A. thaliana* as a heterologous system for dissecting functional and allelic variation in crop genes, providing an efficient alternative to transformation in complex or recalcitrant species such as common bean. Moreover, our results position PvOFP5 as a central regulator of seed development in common bean and highlight its potential as a breeding target for improving seed traits and overall bean quality.

## CONCLUSION

Our study provides a comprehensive resource of QTL and candidate genes controlling leaf and seed morphology and color in common bean, thereby significantly enriching the genetic framework available to the legume research community. By exploring multiple morphological and colorimetric descriptors—many of which were analyzed by GWAS for the first time—we uncovered novel loci and trait relationships that deepen our understanding of phenotypic diversification and its molecular underpinnings. The inclusion of both SNP‐ and SV‐based association analyses proved particularly powerful, enabling the discovery of candidate genes that may have remained undetected by conventional SNP‐only approaches. These findings not only advance fundamental insights into organ development, pigmentation, and adaptation but also provide valuable targets for marker‐assisted selection and genomic prediction in breeding programs. Furthermore, our successful use of *A. thaliana* as a heterologous system to functionally validate PvOFP5 underscores the versatility of this model plant for rapid gene characterization across species. Together, this integrative approach—linking population genomics, quantitative genetics, and functional validation—illustrates an effective and scalable framework for elucidating the genetic architecture of complex traits in crops with long generation times and limited transformation efficiency.

## EXPERIMENTAL PROCEDURES

### Plant material and sequencing

The original seed and sequencing data for the common bean accessions used in this study were previously described (Table S1) (Bellucci et al., 2023; Cortinovis et al., 2024). In total 434 *P. vulgaris* accessions were sequenced by GBS revealing 4792 SNPs, a subset of 200 accessions were selected and sequenced by WGS revealing 7100 duplications and deletions and 3 570 040 SNPs. As reference genome we used the *P. vulgaris* Phavu.G19833v2.1 (common bean genotype G19833) from Phytozome (DOE‐JGI and USDA‐NIFA; https://phytozome‐next.jgi.doe.gov/info/Pvulgaris_v2_1), which comprises approximately 537 Mb assembled into 11 chromosomes with ~27 000 annotated genes. Two experiments were conducted to characterize the morphological traits of leaves and seeds. In Experiment I, 200 WGS accessions were grown under long‐day conditions. Specifically, seeds of each accession were scarified and germinated at room temperature in Petri dishes. Once the radicle had emerged, germinated seeds were transplanted into 30 cm (diameter) × 30 cm (height) pots in a climate‐controlled greenhouse. The plants were grown under an 8/16 h light/dark regime with a photon flux density of 400–500 μmol m^−2^ sec^−1^ (LED lighting), 70% relative humidity, and day/night temperatures of 24°C/20°C, following a randomized complete block design with three biological replicates per accession. In Experiment II, 434 GBS accessions, including the 166 WGS accessions, including three replicates were grown in a polytunnel greenhouse between June and August 2023.

### Morphological analysis

For colorimetric features of seeds and leaves X‐Rite ColorMunki spectrophotometer controlled by ArgyllCMS v.1.9.2 profiling software was utilized. Shape‐related features were analyzed by Adope Photoshop® and Python 3.6 ANACONDA distribution using ‘skimage’, ‘pandas’, and ‘matplotlib’. We summarized the wavelength detected by ‘ColorMunki’ spectrophotometer to the color ranges of the visible light (violet: 380–450 nm, blue: 450–495 nm, green: 495–570 nm, yellow: 570–590 nm, orange: 590–620 nm, red: 620–750 nm). ‘Spectra Integral’ is the total reflectance over the full spectral range measured and gives an indication of brightness or reflectance intensity, ‘520/580’ is referred to as the reflectance in green‐yellow range between 520 and 580 nm and indicates the chlorophyll or carotenoid content, ‘Chlorophyll’ is the chlorophyll‐specific index (670 and 750 nm), and ‘Infrared’ the infrared reflectance (700–1000 nm) often used to describe plant health. The red, green and blue channel intensities of the visible spectrum is described as ‘R’/‘G’/‘B’, the HSV model with hue (type of color), saturation (intensity/purity of color), brightness (value/luminance, color lightness or darkness) with ‘H’/‘S’/‘BV’, while ‘X’/‘Y’/‘Z’ is the color space components CIE 1931 tristimulus values. The CIELAB color refers to ‘L’/‘a’/‘b’ and is the lightness, red‐greenness, and yellow‐blueness. For the seed 434 panel the gray value which is a proxy for the brightness level of individual pixels and the integrated density which is the sum of all pixel values in a region was calculated.

As morphological descriptors, the area, extent (bounding box area; shape compactness versus bounding box), solidity (convex hull area; convexity; 1 = solid, <1 = concave), perimeter, eccentricity (ellipticity − shape elongation), centroid (geometric center of the object), roundness (4·*π*·area/perimeter^2^), circularity (roundness measure), size, height, width, and weight were considered.

### GWAS of morphological traits

For GWAS, the R package GAPIT3 (Wang & Zhang, 2021) was used, employing EMMA (efficient mixed‐model association) as a variance component method to correct for population structure effects with a kinship matrix and three PCs as fixed effects. MLM, compressed MLM, MLMM (Zhang et al., 2010), BLINK (Huang et al., 2019), and FarmCPU (Liu et al., 2016) were used as models for association testing to identify significant associations between genetic variants (SNPs) and the phenotype of interest. We used the Bonferroni correction to adjust the significance threshold for multiple comparisons and used only signals detected by up to three methods for downstream analysis. Overlaid Manhattan plots were generated in the R package rMVP (Yin et al., 2021). Candidate genes were identified by scanning the genetic area ± 100 kb of the lead SNP.

### Genomic analysis

For the analysis of the genomic diversity, Tassel 5 (Bradbury et al., 2007) was used in combination with iTOL (https://itol.embl.de/) for visualization of the WGS phylogenetic tree. To assess population structure within the GBS GWAS panel, we used STRUCTURE v2.3.4 (Pritchard et al., 2000) under the admixture model with correlated allele frequencies, testing *K* values from 1 to 13. For each K, six independent runs were performed, each with 500 burn‐in iterations followed by 5000 Markov Chain Monte Carlo iterations. The optimal number of clusters was determined using the Δ*K* (Evanno et al., 2005) method implemented in PopHelper (Francis, 2017). Accessions with an unknown origin were assigned to either the Mesoamerican or Andean gene pool (membership ≥0.8), or classified as admixed if the percentage of membership of any sub‐cluster was <0.8.

### Data analysis

Data were analyzed using the R environment version 4.3.2 using the packages ‘ggplot2’. Significances were computed by either anova with *post‐hoc* Tukey HSD test or Kruskal–Wallis and *post‐hoc* Dunn‐test based on the data normal distribution.

### Network analysis

The morphological and colorimetric data network was constructed with features annotated by tissue and class. Pairwise Pearson correlations between metabolites were calculated, and edges were retained for correlations |*r*| >0.7 with *P* < 0.05. The network was built using ‘igraph’, with node degree calculated to identify hub features. The network was visualized using ‘ggraph’ with the Kamada‐Kawai layout. All analyses were performed in R (4.3.2.) using the ‘tidyverse’, ‘igraph’, and ‘ggraph’ packages.

### Genotyping of *A. thaliana* T‐DNA insertion lines

DNA was extracted following (Berendzen et al., 2005) from SALK_201425 and SALK_203823, and positive T‐DNA insertion lines were identified following the instructions of the provider (http://signal.salk.edu/tdnaprimers.2.html).

### *Arabidopsis thaliana* OX and complementation

The total RNA from ECe122 (MES, small seeds; OFP5‐1) and ECe153 (AND, large seeds; OFP5‐2) was isolated using a NucleoSpin® RNA plant kit (Macherey‐Nagel, Düren, Germany) according to the manufacturer's instructions. First‐strand cDNA was synthesized using 1.5 μg RNA and Prime Script™ RT reagent Kit with gDNA eraser (Takara, Kusatsu, Japan) according to the manufacturer's instructions. Full‐length cDNA of leaf samples of the founder lines was amplified by using 250 ng (fw‐primer: TAGGTACCATGGAACCAAGAAGGAACAC, rv‐primer: TATACTCGAGAGTCGCCTCCACTGACAAG). Gateway™ pENTR™ 1A Dual Selection Vector was used for restriction‐enzyme based cloning (KpnI and XhoI) followed by LR recombination using pK2GW7 (Karimi et al., 2002). Columbia‐0 and T‐DNA insertion lines of *OFP5* were transformed using *Agrobacterium tumefaciens* strain GV3101 (Zhang, Chen, et al., 2020) and screened for single homozygous insertions using kanamycin resistance.

### *Arabidopsis thaliana* phenotyping

Plants were grown under long‐day conditions in a greenhouse and phenotyped for rosette diameter and plant height. For seed size and silique measurements, seeds were placed on a square‐shaped Petri dish, while siliques were scanned directly without support (Figure S10). All samples were scanned at a resolution of 600 dpi. Seed area [mm^2^], width and length [mm] and silique roundness, length [cm] and width [mm] was quantified using Python 3.6 (Anaconda distribution) with the ‘skimage’, ‘pandas’, and ‘matplotlib’ library.

## AUTHOR CONTRIBUTIONS

SA conceptualized the experiment. JvS and SA wrote the manuscript with input from all authors. ARF provided guidance on experimental strategy. AP, GF, LN, EBe, EBi, and RP provided the germplasm and the sequencing data. SA, GP, and MB imaged seeds and leaves. GP performed seed and leaves spectral measurements. JvS, MB, SB, RW, YS, FZ performed image analysis. JvS and MB performed GWAS. JvS performed data analysis, visualization, and gene validation.

## CONFLICT OF INTEREST

The authors declare no conflict of interest.

## Supporting information


**Table S1.** Information on common bean accessions used in this study.
**Table S2.** Morphological and colorimetric descriptors of leaves and seeds.
**Table S3.** Clusters and features of network analysis of morphological and colorimetric leaf and seed descriptors.
**Table S4.** Mapping results of genome‐wide association study (GWAS) using single‐nucleotide polymorphisms (SNPs) of genotyping‐by‐sequencing and whole‐genome sequencing or structural variants of leaf color, morphology, and seed color and morphology.
**Table S5.** Genotyping‐by‐sequencing significant associations of ≥ two methods of genome‐wide association study using leaf and seed morphology and colorimetric data.
**Table S6.** Significant structural variants of ≥ two methods of genome‐wide association study using leaf and seed morphology and colorimetric data.
**Table S7.** Whole‐genome sequencing significant associations of ≥ two methods of genome‐wide association study using leaf and seed morphology and colorimetric data.
**Table S8.** Novel candidate genes identified using seed and leaf morphological and colorimetric descriptors with GBS, WGS‐SV, and WGS‐SNP data.
**Table S9.** GWAS results for seed area in the full panel of 434 accessions genotyped by genotyping‐by‐sequencing, analyzed using MLM, CMLM, MLMM, BLINK, and FarmCPU models.

**Figure S1.** Methodological overview.
**Figure S2.** Population structure analysis of common bean diversity panel.
**Figure S3.** Colorimetric features of leaves of the subset of 200 accessions.
**Figure S4.** Morphological features of leaves of the subset of 200 and the full 434 panel.
**Figure S5.** Colorimetric and morphological features of seeds of the subset of 200 accessions.
**Figure S6.** Colorimetric and morphological features of seeds of the subset of 434 accessions.
**Figure S7.** Genome‐wide association mapping of leaf and seed agro‐morphological traits using MLM, CMLM, MLMM, BLINK, and FarmCPU.
**Figure S8.** Significant associations across five GWAS methods using GBS, SV, and WGS data.
**Figure S9.** *AtOFP5* T‐DNA insertion lines *ofp5_1* and *ofp5_2*.
**Figure S10.** High‐throughput phenotyping of siliques and seeds of *Arabidopsis thaliana*.

## Data Availability

The authors declare that the data supporting the findings of this study are available within the paper and its Supporting Information files.
