# Supplementary material for: GWAS identifies regulators of seed and leaf morphology in common bean, revealing OFP5 as a major seed size determinant
Source: Plant J. 2026 May 6;126(3):e70909. doi: 10.1111/tpj.70909 (PMC13147493; doi:10.1111/tpj.70909)
Supplement: Supplementary file 2 — Figure S1. Methodological overview. Figure S2. Population structure analysis of common bean diversity panel. Figure S3. Colorimetric features of leaves of the subset of 200 accessions. Figure S4. Morphological features of leaves of the subset of 200 and the full 434 panel. Figure S5. Colorimetric and morphological features of seeds of the subset of 200 accessions. Figure S6. Colorimetric and morphological features of seeds of the subset of 434 accessions. Figure S7. Genome‐wide association mapping of leaf and seed agro‐morphological traits using MLM, CMLM, MLMM, BLINK, and FarmCPU. Figure S8. Significant associations across five GWAS methods using GBS, SV, and WGS data. Figure S9. AtOFP5 T‐DNA insertion lines ofp5_1 and ofp5_2. Figure S10. High‐throughput phenotyping of siliques and seeds of Arabidopsis thaliana. [file TPJ-126-0-s001.pdf]

Supplementary Figures

GWAS identifies regulators of seed and leaf morphology in common bean, revealing OFP5 as a major seed size determinant

Julia von Steimker[1], Mustafa Bulut[1,2], Gregory Pozhvanov[1,3], Susan Bergmann[1], Regina Wendenburg[1], Yuming Sun[4], Feng Zhu[5], Alice Pieri[6], Giulia Frascarelli[6], Laura Nanni[6], Elisa Bellucci[6], Elena Bitocchi[6], Roberto Papa[6], Alisdair R. Fernie[1,7], Saleh Alseekh[1,7*]

[1] Max-Planck-Institute of Molecular Plant Physiology, Am Mühlenberg 1, 14476, Potsdam-Golm, Germany

[2] Program Center MetaCom, Leibniz Institute of Plant Biochemistry, Weinberg 3, Halle (Saale) 06120, Germany

[3] Komarov Botanical Institute, Russian Academy of Sciences, Saint-Petersburg, Russia

[4] Jiangsu Key Laboratory for Conservation and Utilization of Plant Resources, Institute of Botany, Jiangsu Province and Chinese Academy of Sciences, Nanjing 210014, China

[5] National R&D Center for Citrus Preservation, Key Laboratory of Horticultural Plant Biology, Ministry of Education, Huazhong Agricultural University, Wuhan 430070, China

[6] Department of Agricultural, Food and Environmental Sciences, Polytechnic University of Marche, Via Brecce Bianche, 60131 Ancona, Italy

[7] Center of Plant Systems Biology and Biotechnology, 4000 Plovdiv, Bulgaria

*Corresponding author (alseekh@mpimp-golm.mpg.de)

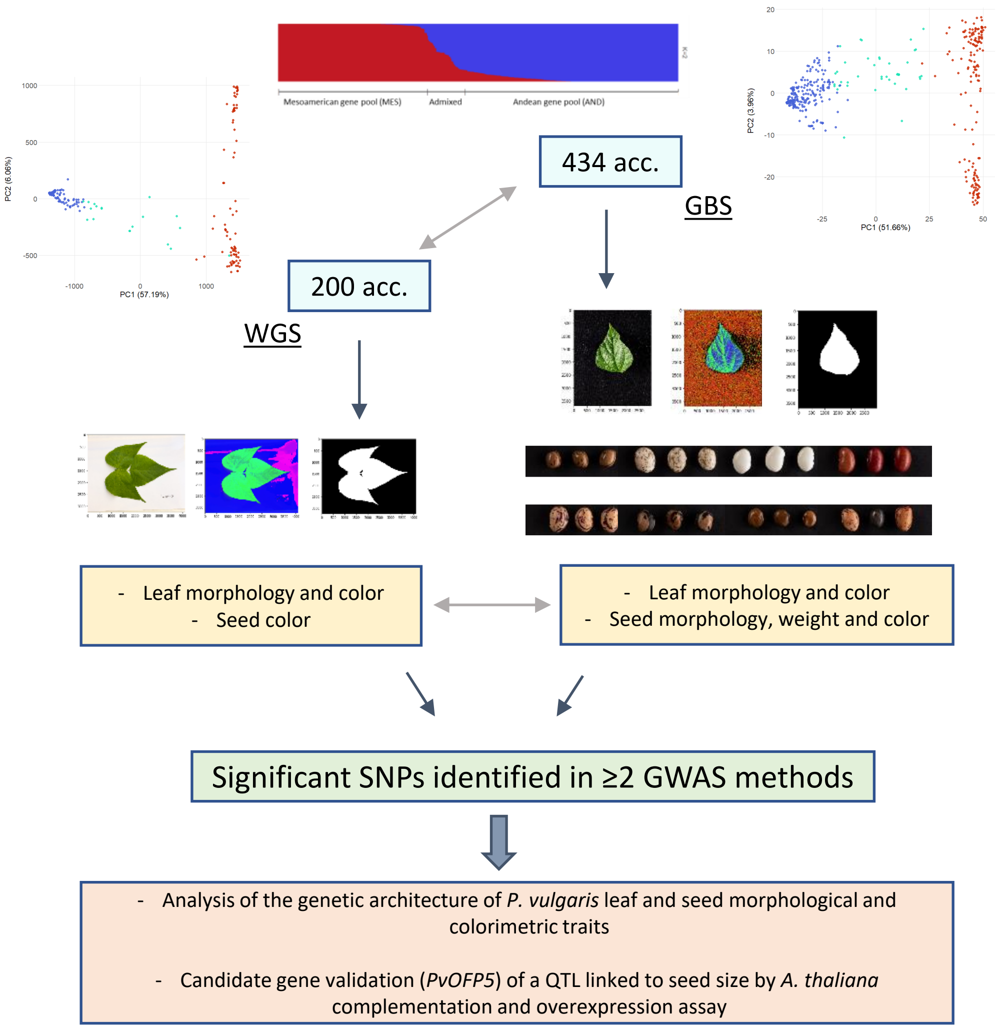

Figure S1: Methodological overview. In total 434 and 200 accessions (acc.) were sequenced using genotyping-by-sequencing (GBS) and whole-genome sequencing (WGS), respectively. Principal component analysis (PCA) of single-nucleotide polymorphism data was conducted using the gene pool as group (Mesoamerican (MES, red), admixed (turquoise), Andean (AND, blue)). Leaf images were segmented and morphological traits calculated via Python, while seed mnorphological descriptors were obtained using Adope Photoshop®. Colorimetric parameters were obtained using 'Colormunki'. Data were subjected to five GWAS methods (CMLM, MLMM, MLM, FarmCPU and BLINK). Only significant SNPs identified in at least 3 methods were taken into consideration for the analysis of the genetic architecture of *P. vulgaris*. The candidate gene *PvOFP5* was functionally validated for seed size in *A. thaliana* complementation and overexpression lines.

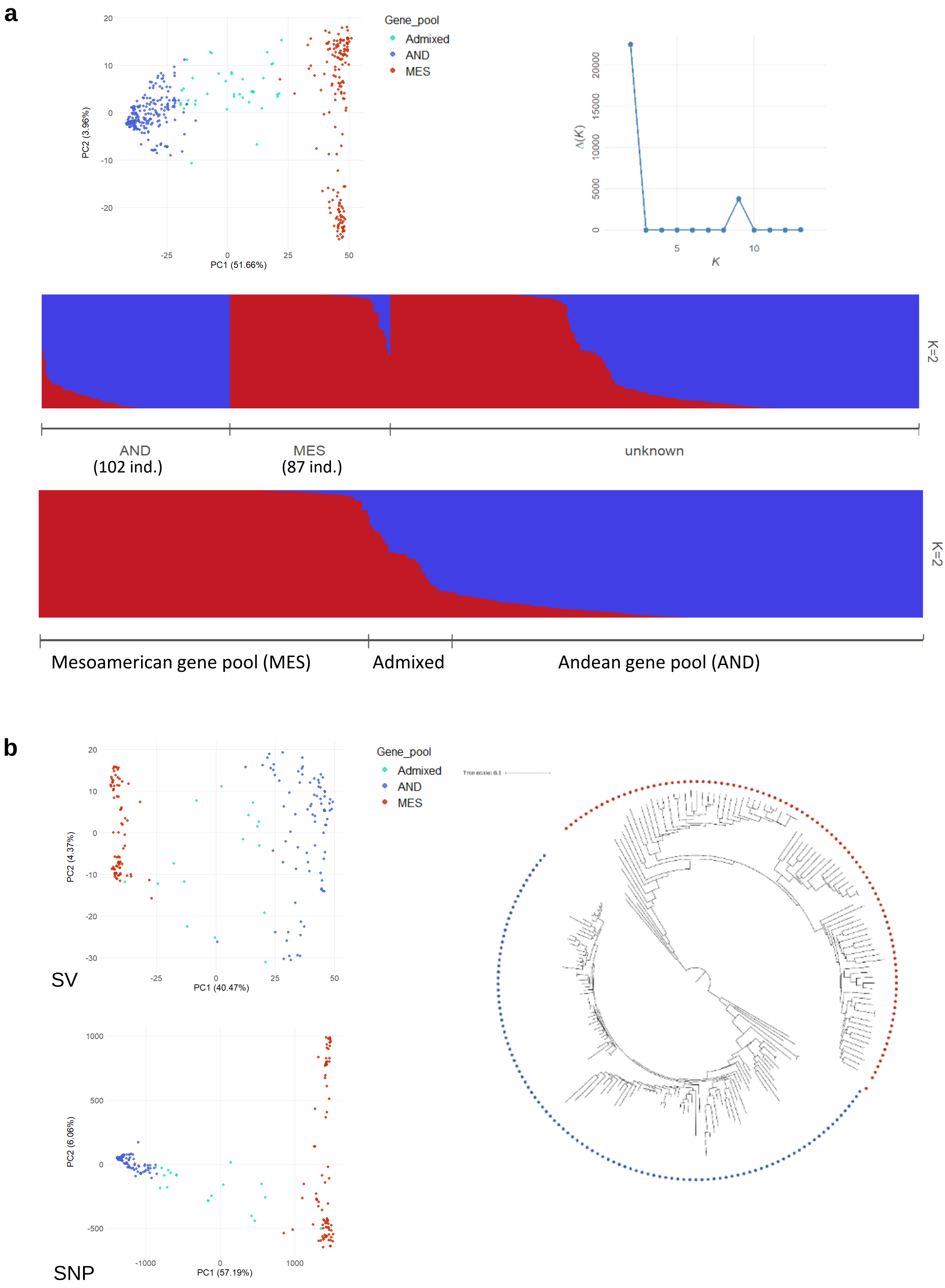

Figure S2: Population structure analysis of common bean diversity panel. a) Principal component analysis (PCA) and hierarchical population structure analysis of 434 accessions of *P. vulgaris* using 4,771 genotyping-by-sequencing (GBS) single-nucleotide polymorphisms (SNP). The number of subpopulations was most likely inferred at K = 2. Population structure at K = 2 based on Q matrix assigning accessions to the Mesoamerican and Andean gene pool. Colors represent subpopulations. b) PCA of 200 accessions using structural variants (SV) and SNP data obtained by whole-genome-sequencing (WGS). Neighbour joining tree of 200 accessions using WGS SNP data.

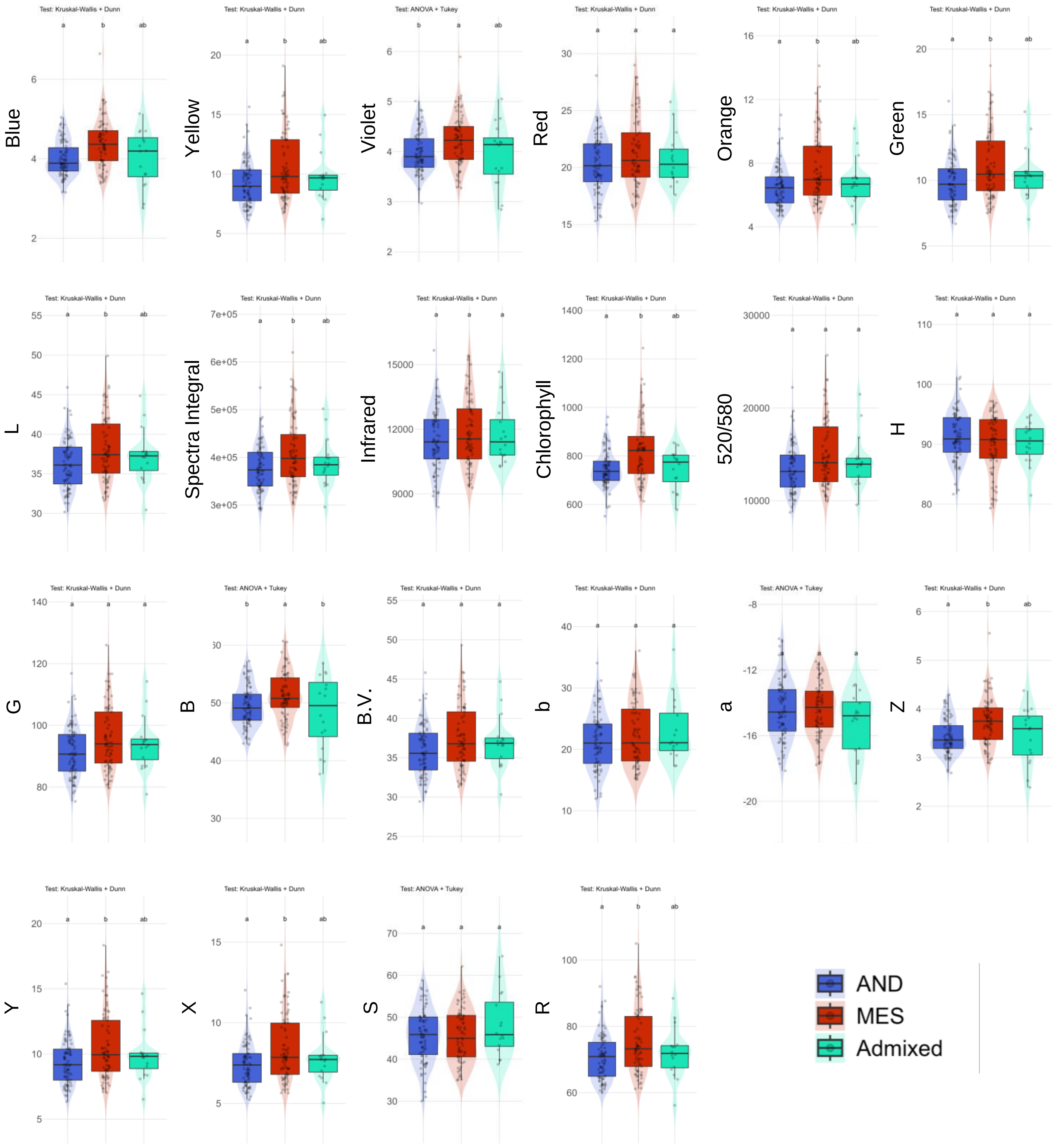

Figure S3: Colorimetric features of leaves of the subset of 200 accessions. Boxplots of the leaf 200 WGS panel grouped by Andean (AND), Mesoamerican (MES) gene pool and admixed. Color ranges of the visible light (violet: 380 – 450 nm, blue: 450 – 495 nm, green: 495 – 570 nm, yellow: 570 – 590 nm, orange: 590 – 620 nm, red: 620 – 750 nm). ‘Spectra Integral’ is the total reflectance over the full spectral range measured and gives an indication of brightness or reflectance intensity, ‘520/580’ is referred to as the reflectance in green-yellow range between 520 and 580 nm and indicates the chlorophyll or carotenoid content, ‘chlorophyll’ is the chlorophyll-specific index (670 and 750 nm), and ‘Infrared’ the infrared reflectance (700 – 1000 nm) often used to describe plant health. The red, green and blue channel intensities of the visible spectrum is described as ‘R’/’G’/’B’, the HSV model with hue (type of color), saturation (intensity/purity of color), brightness (value/luminance, color lightness or darkness) with ‘H’/’S’/’BV’, while ‘X’/’Y’/’Z’ is the color space components CIE 1931 tristimulus values. The CIELAB color refers to ‘L’/’a’/’b’ and is the lightness, red-greenness and yellow-blueness. Letters indicate significance calculated with either Kruskal-Wallis post-hoc Dunn-test or ANOVA post-hoc Tukey HSD test as indicated based on the data normal distribution.

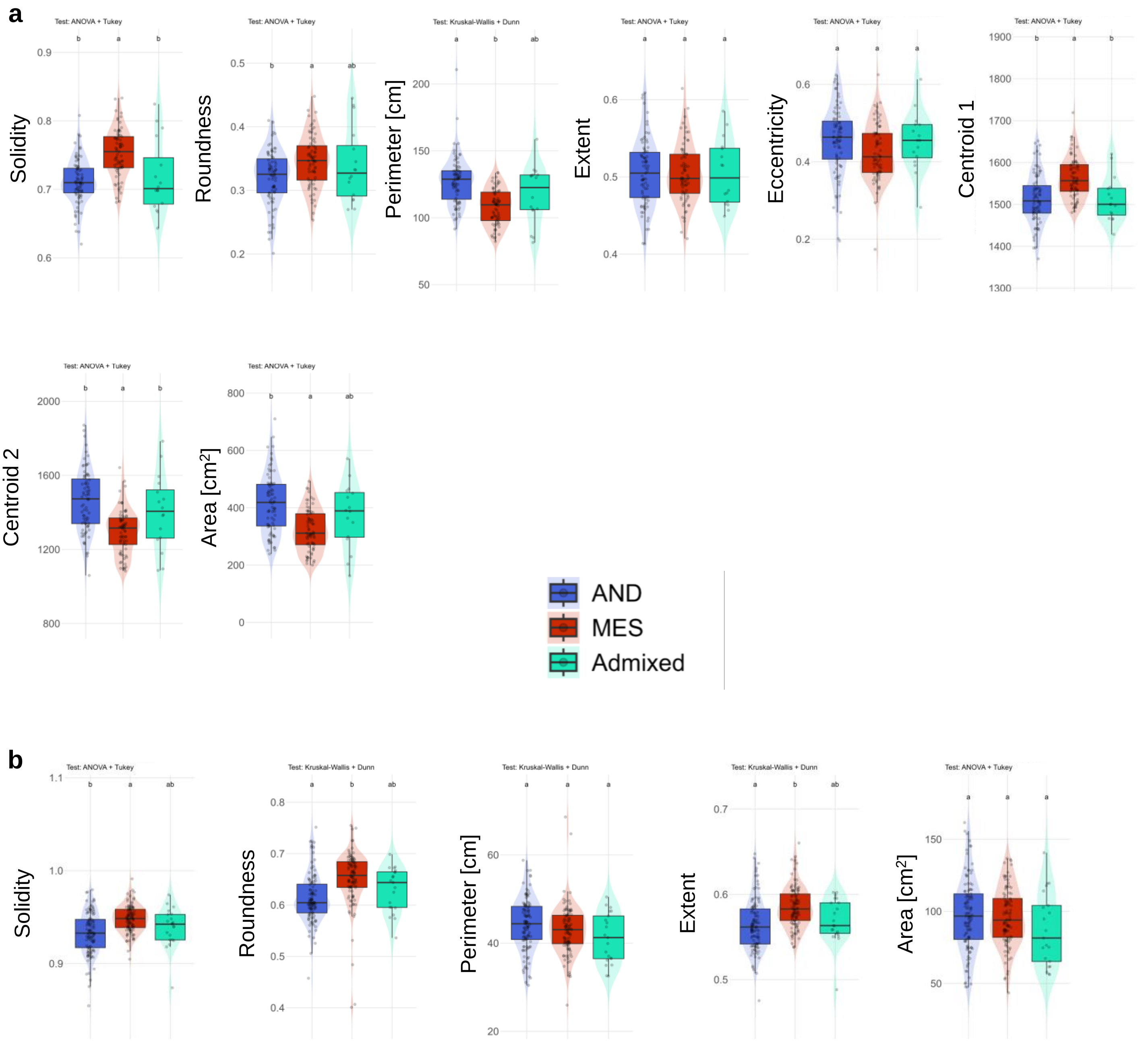

Figure S4: Morphological features of leaves of the subset of 200 and the full 434 panel. Boxplots of the a) leaf 200 WGS panel and the b) 434 GBS panel grouped by Andean (AND), Mesoamerican (MES) gene pool and admixed. As morphological descriptors the area in cm^2, extent (bounding box area; shape compactness vs. bounding box), solidity (convex hull area; convexity; 1 = solid, <1 = concave), perimeter in cm, eccentricity (ellipticity – shape elongation), centroid (geometric center of the object), roundness, and circularity (roundness measure) were considered. Letters indicate significance calculated with either Kruskal-Wallis post-hoc Dunn-test or ANOVA post-hoc Tukey HSD test as indicated based on the data normal distribution.

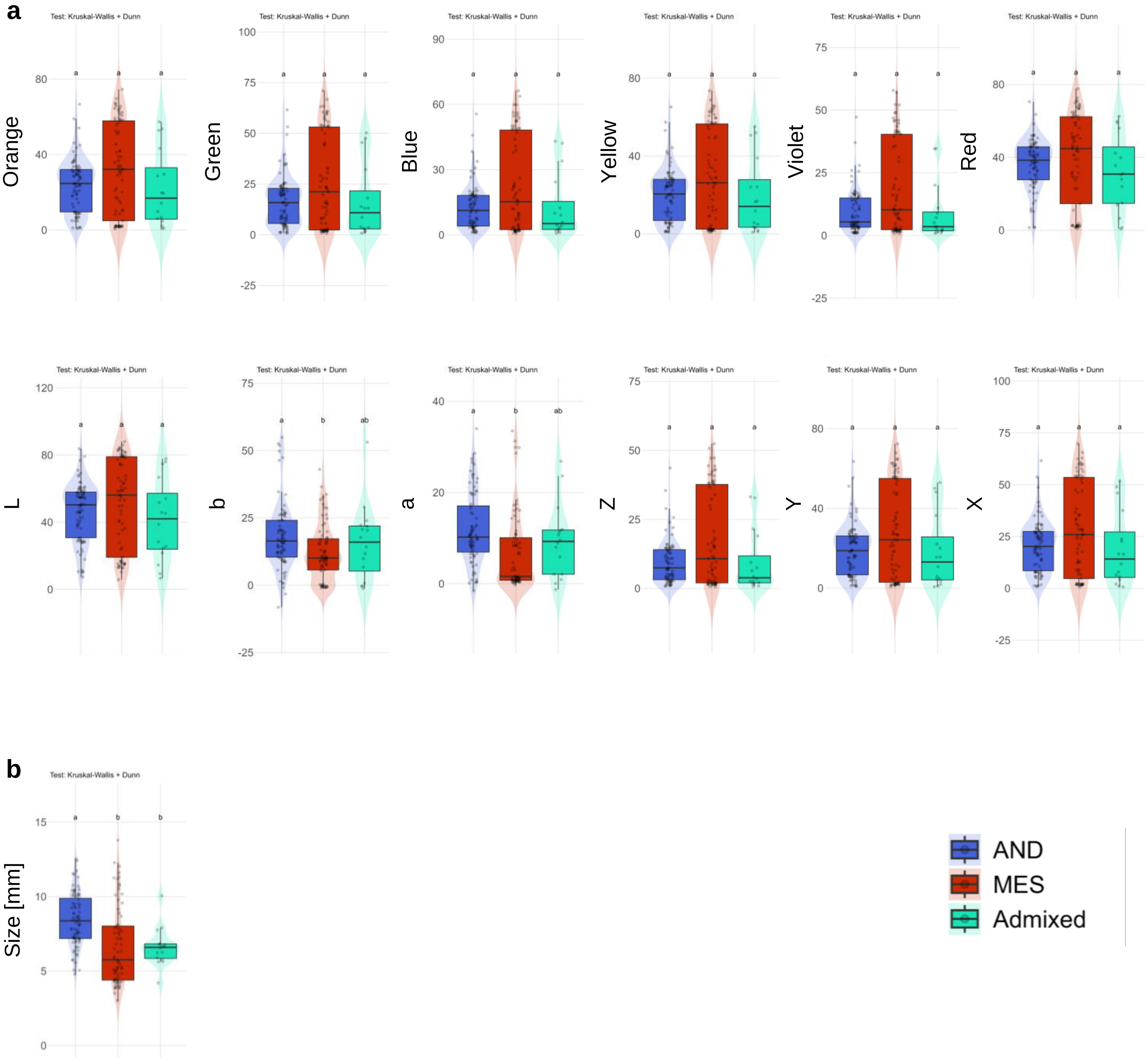

Figure S5: Colorimetric and morphological features of seeds of the subset of 200 accessions. Boxplots of the seed 200 WGS panel grouped by Andean (AND), Mesoamerican (MES) gene pool and admixed. a) Color ranges of the visible light (violet: 380 – 450 nm, blue: 450 – 495 nm, green: 495 – 570 nm, yellow: 570 – 590 nm, orange: 590 – 620 nm, red: 620 – 750 nm). ‘X’/’Y’/’Z’ is the color space components CIE 1931 tristimulus values and the CIELAB color refers to ‘L’/’a’/’b’ and is the lightness, red-greenness and yellow-blueness. b) Seed size in mm. Letters indicate significance calculated with either Kruskal-Wallis post-hoc Dunn-test or ANOVA post-hoc Tukey HSD test as indicated based on the data normal distribution.

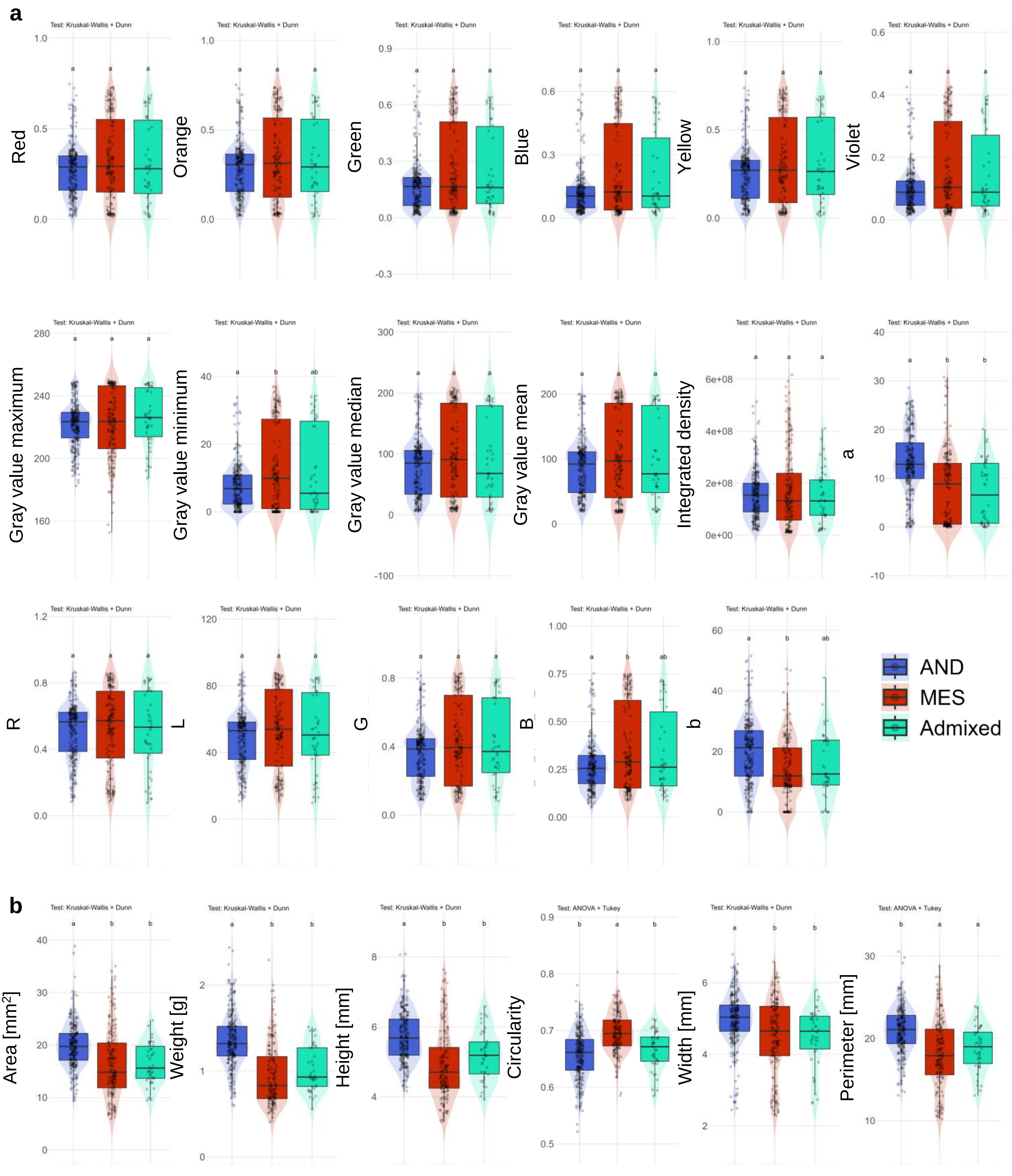

Figure S6: Colorimetric and morphological features of seeds of the subset of 434 accessions. Boxplots of the seed 200 WGS panel grouped by Andean (AND), Mesoamerican (MES) gene pool and admixed. a) Color ranges of the visible light (violet: 380 – 450 nm, blue: 450 – 495 nm, green: 495 – 570 nm, yellow: 570 – 590 nm, orange: 590 – 620 nm, red: 620 – 750 nm). The red, green and blue channel intensities of the visible spectrum is described as ‘R’/’G’/’B’, and the CIELAB color refers to ‘L’/’a’/’b’ and is the lightness, red-greenness and yellow-blueness. The gray value is a proxy for the brightness level of individual pixels and the integrated density is the sum of all pixel values in a region. b) Seed area in mm^2, weight in g, height, width, perimeter in mm and circularity. Letters indicate significance calculated with either Kruskal-Wallis post-hoc Dunn-test or ANOVA post-hoc Tukey HSD test as indicated based on the data normal distribution.

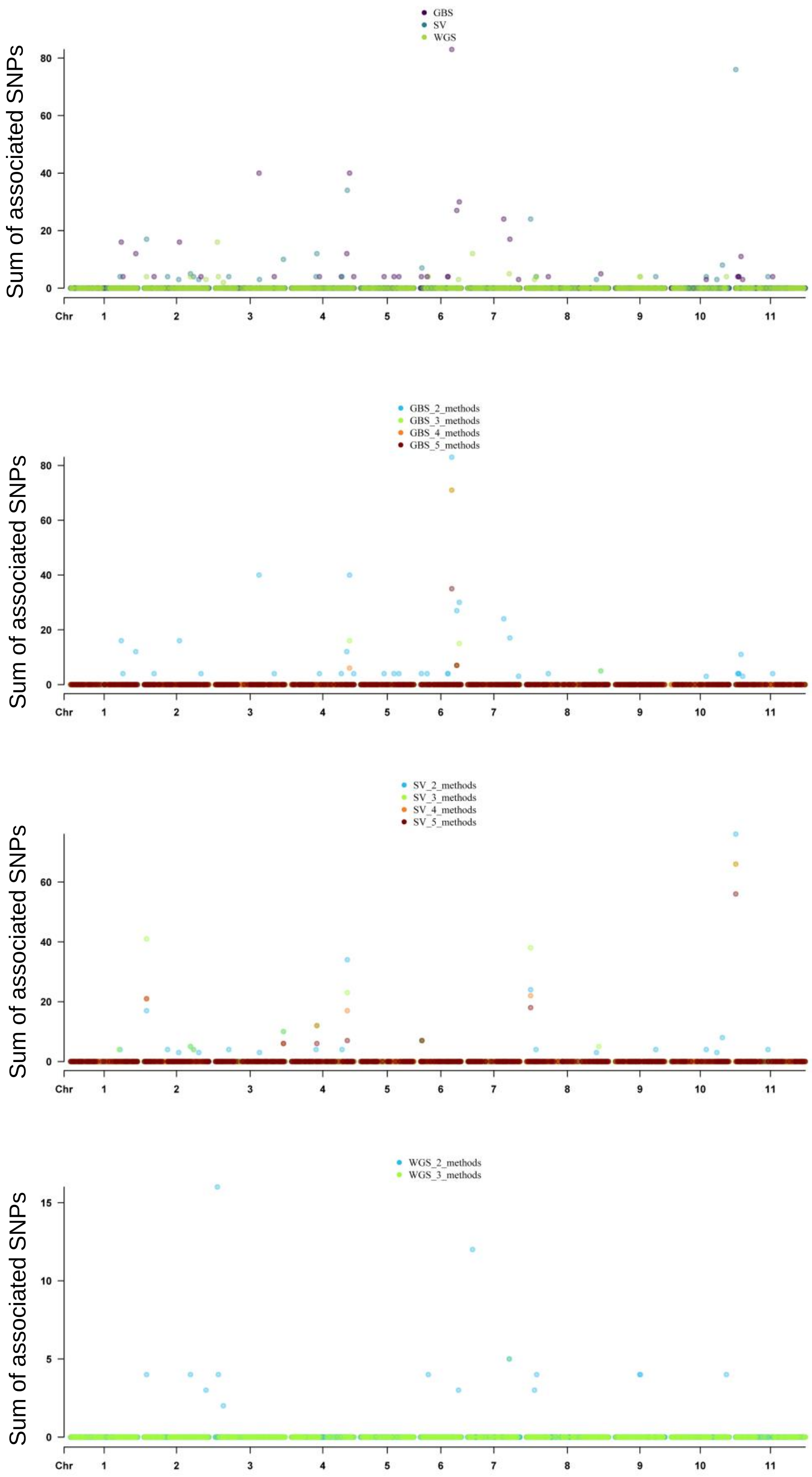

Figure S7: Genome-wide association mapping of leaf and seed agro-morphological traits using MLM, CMLM, MLMM, BLINK and FarmCPU. Manhattan plot of the counted number of associated traits with a genomic association found in either all five methods (red), four (orange), three (green) or two (blue). For the mapping either genotyping-by-sequencing (GBS), structural variance (SV) or whole-genome sequencing (WGS) data were used.

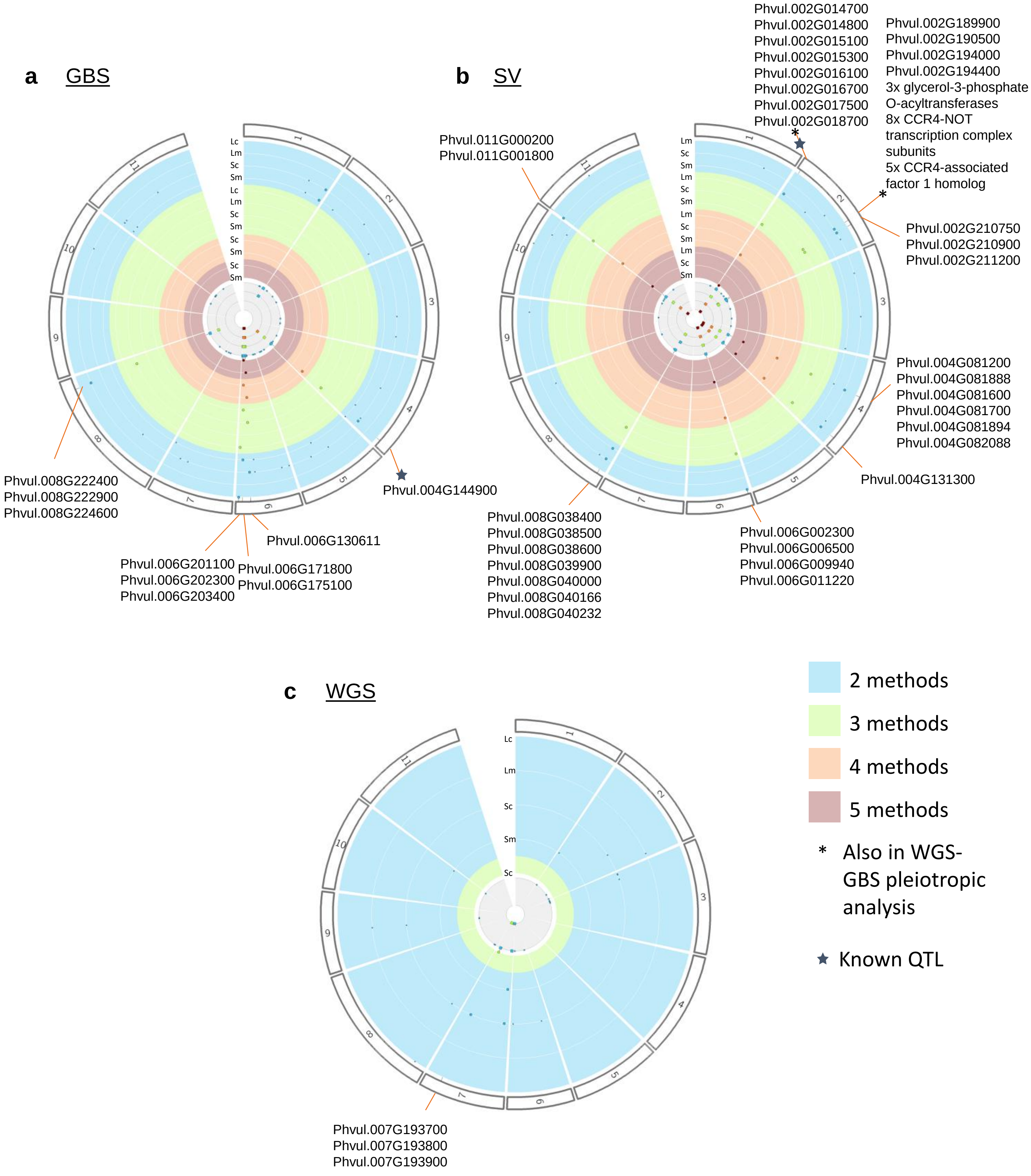

Figure S8: Significant associations across five GWAS methods using GBS, SV and WGS data. Counts of associated traits of leaf and seed color and morphology using a) genotype-by-sequencing (GBS) data, b) structural variance (SV) data and c) whole-genome sequencing (WGS) data. Common QTL across all methods (BLINK, CMLM, MLM, MLMM, FarmCPU) are highlighted with a candidate gene. Lc = leaf color traits, Lm = leaf morphology traits, Sc = Seed color traits, Sm = Seed morphology traits.

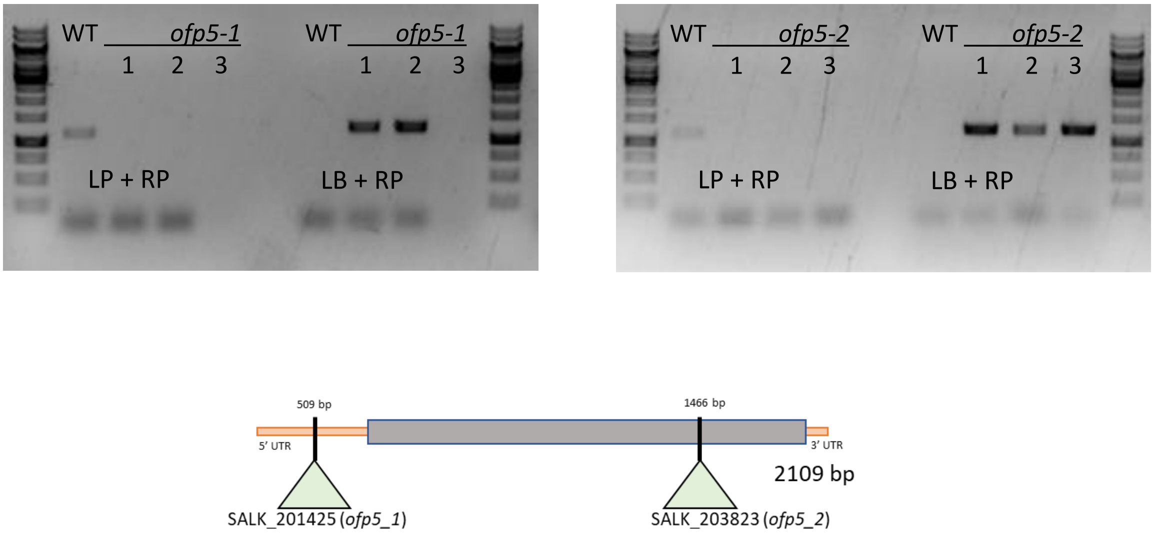

Figure S9: AtOFP5 T-DNA insertion lines *ofp5_1* and *ofp5_2*. *Arabidopsis thaliana* SALK T-DNA insertion lines of OFP5 were genotyped using LP plus RP and LB plus RP primers.

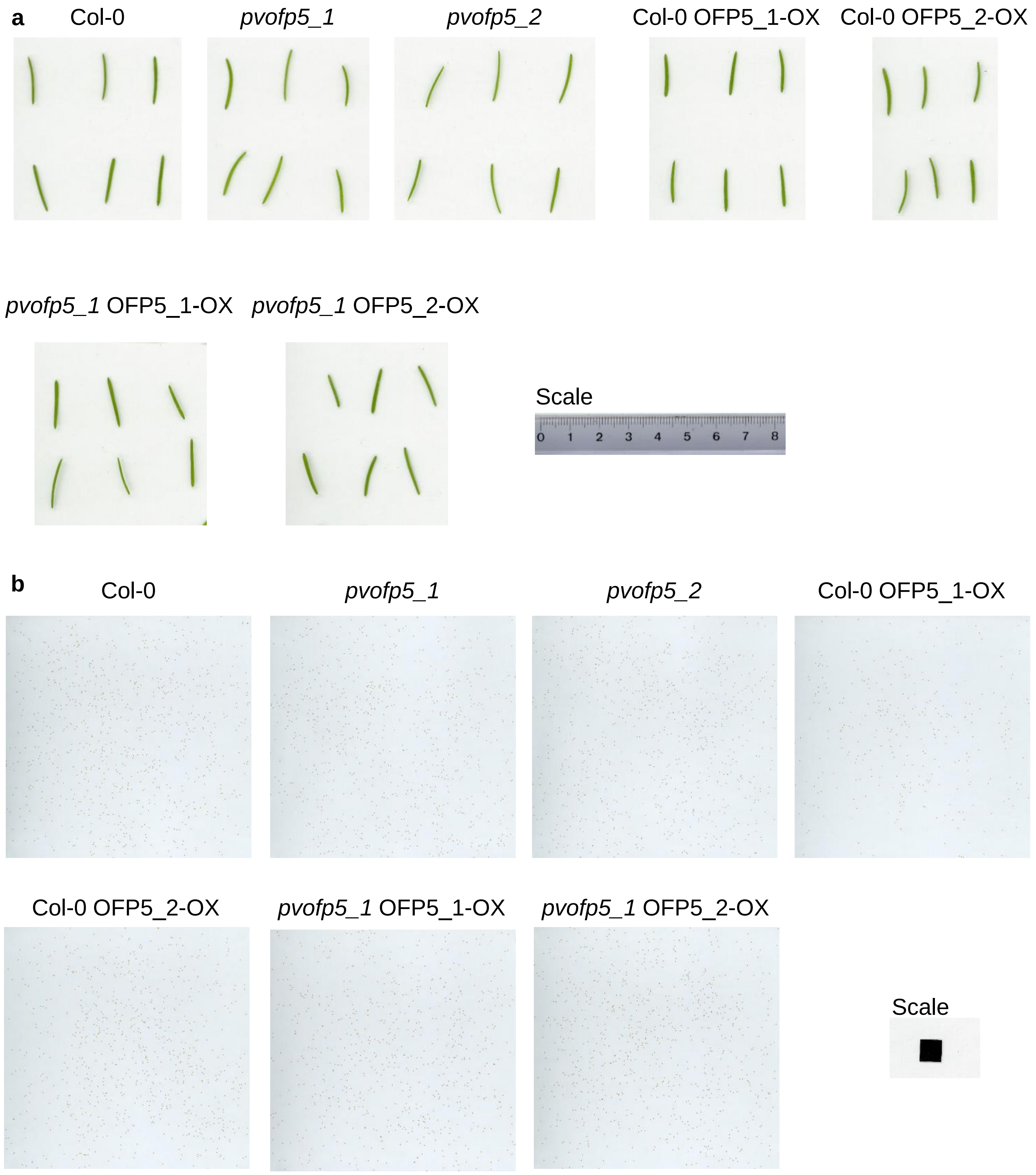

Figure S10: High-throughput phenotyping of siliques and seeds of *Arabidopsis thaliana*. For image analysis a) siliques and b) seeds were scanned using a flatbed scanner using a) a ruler or b) a 1 cm^2 square. Columbia-0 (Col-0) was used as wild type (WT), pvofp5_1 and pvofp5_2 are two independent knock out lines, Col-0/*pvofp5_1* OFP5_1-OX is the inferior allele in WT or mutant background, Col-0/*pvofp5_1* OFP5_2-OX is the superior allele in WT or mutant background.
